# Development and validation of a food retail environment analysis protocol in Iran

**DOI:** 10.1017/S1368980025100773

**Published:** 2025-08-05

**Authors:** Mohammadhassan Rostami, Mina Babashahi, Arezoo Rezazadeh, Nasrin Omidvar, Hamed Zamanpoor

**Affiliations:** 1 Student Research Committee, School of Nutrition and Food Sciences, Shiraz University of Medical Sciences, Shiraz, Iran; 2 Nutrition Research Center, Shiraz University of Medical Sciences, Shiraz, Iran; 3 Department of Community Nutrition, School of Nutrition and Food Sciences, Shiraz University of Medical Sciences, Shiraz, Iran; 4 Department of Community Nutrition, National Nutrition and Food Technology Research Institute, Faculty of Nutrition Sciences and Food Technology, Shahid Beheshti University of Medical Sciences, Tehran, Iran; 5 Department of Community Nutrition, Faculty of Nutrition Sciences and Food Technology, National Nutrition and Food Technology Research Institute, Shahid Beheshti University of Medical Sciences, Tehran, Iran; 6 Department of Exercise Physiology, Faculty of Physical Education and Sport Sciences, University of Tehran, Tehran, Iran

**Keywords:** Food environment, INFORMAS, Protocol, Retail, Validation

## Abstract

**Objective:**

This study aimed to adapt and validate a Food Retail Environment Analysis Protocol in Shiraz, Iran.

**Design:**

The protocol was developed by integrating the Nutrition Environment Measurement Survey in Stores with the food retail module from the International Network for Food and Obesity/Non-communicable Diseases Research, Monitoring, and Action Support. After translating, synthesising and back-translating the protocol, a panel of experts reviewed and refined it to ensure cultural and contextual appropriateness. Its validity was assessed through expert evaluation, and the pre-final version was field-tested to assess reliability across different food retail environments.

**Setting:**

Shiraz City, a metropolis in Iran

**Participants:**

Nine food retail stores, including kiosks, small- and medium-sized food retailers (comparable to convenience stores) and large food retailers (comparable to grocery stores).

**Results:**

Content and face validity were assessed using the content validity ratio (0·64–1), content validity index (0·78–1) and item impact score (2·84–4·83). Reliability testing by two researchers showed a 93·77 % agreement and an intraclass correlation coefficient of 0·89–1. The protocol includes fourteen food groups, most of which are categorised as either healthy or unhealthy. It evaluates product availability, prominence, quality, pricing and both in-store and out-of-store food promotions.

**Conclusion:**

The validated protocol effectively assesses diverse retail food environments, offering essential data for evaluating policies and guiding interventions to improve healthy food access. It is adaptable for broader regional or international application in public health and food policy contexts.

The global food supply has increasingly shifted towards processed and ultra-processed foods, which are now widely available, affordable and heavily marketed^(1–5)^. The convenience of these products, which require minimal preparation, has significantly contributed to their increased consumption^(5)^. However, this shift is associated with major public health issues, including obesity, type 2 diabetes, fatty liver disease and other chronic conditions, ultimately leading to higher healthcare costs^(1,6–11)^.

One of the major global health challenges is overweight and obesity, which now affects not only high-income countries but also low- and middle-income nations^(12)^. As of 2022, one in every eight people worldwide was living with obesity, with 43 % of adults aged 18 and older classified as overweight and 16 % as obese. Since 1990, these rates have more than doubled globally^(12)^. This growing trend is particularly concerning in low- and middle-income countries, including Iran. National data from 2019 indicate that approximately 20·6 % of Iranian adults were overweight, and 14·6 % were obese^(13,14)^. Given the increasing availability and consumption of processed foods, it is essential to focus on food environments and dietary patterns to improve public health outcomes beyond the issue of obesity alone^(15)^.

Food environments play a critical role in shaping dietary behaviours and are thus recognised as a key area for preventive and interventional strategies^(9–11,16)^. One of the primary factors contributing to the rising rates of obesity is the presence of obesogenic food environments – places where unhealthy foods are widely available and heavily marketed^(15,17)^. Several studies have demonstrated a positive association between the density of fast-food outlets and retail stores offering unhealthy food options in a given area and higher rates of overweight and obesity among the population^(16,18)^.

To address these issues, many countries have implemented policies to restrict the availability (i.e. sale and provision) of unhealthy foods as well as to regulate their marketing and promotion^(19–21)^. However, the effectiveness of these policies requires comprehensive tools that can accurately assess the food retail environment and provide reliable data for developing intervention strategies.

The International Network for Food and Obesity/Non-communicable Diseases (NCDs) Research, Monitoring, and Action (INFORMAS) is an international network of public-interest organisations and researchers that aims to monitor, benchmark, and support public and private sector actions to improve healthy food environments and reduce obesity, NCDs and related inequalities. The Retail Food Module within INFORMAS provides a structured approach to evaluating food retail environments by assessing the marketing mix – pricing, product availability, advertising and the presence of foods and beverages. It also examines the spatial distribution of retail stores and offers a step-by-step framework for monitoring supermarkets and other food retailers^(22)^.

Similarly, the Nutrition Environment Measurement Survey for Stores (NEMS-S) was developed in 2007 as a standardised tool for assessing the nutritional quality of food retail environments. This tool focuses on evaluating the availability, quality and variety of foods, particularly in terms of comparing healthier and less healthy options. It collects data across ten food categories based on North American dietary guidelines through direct observation, emphasising high-fat and high-calorie foods. Additionally, NEMS-S incorporates price comparisons and provides reliable, objective data for understanding the impact of food environments on dietary behaviours and health outcomes^(23–25)^. Due to its adaptability and ease of use, NEMS-S has been widely applied across different settings and populations^(26–29)^.

Although several comprehensive tools exist internationally to assess food retail environments, none have been specifically adapted or validated for the Iranian context. To address this gap, the present study combined and tailored two internationally recognised tools to develop a culturally and contextually appropriate protocol for the Iranian setting. Specifically, the broad, policy-oriented monitoring approach of the INFORMAS Retail Food Module was integrated with the detailed, in-store assessment framework of NEMS-S. The combined protocol ensures a more comprehensive evaluation of the food environment by capturing both macro- and micro-level indicators – ranging from the geographical distribution of outlets and availability of food items to in-store promotion and pricing practices. This approach enhances the protocol’s applicability for policy evaluation and intervention planning while ensuring alignment with international methodologies and adaptability to the local Iranian context.

Given the urgent need for local data to inform policymaking and intervention strategies, this study aimed to develop and validate a culturally adapted protocol for assessing food retail environments in Iran, using Shiraz (a city in central Iran) as a representative urban setting. Developing a comprehensive tool tailored to the local context can contribute to improving food environments and supporting public health initiatives aimed at promoting healthy dietary patterns and reducing the burden of non-communicable diseases.

## Methodology

This study employed a structured protocol to evaluate the retail food environment in urban regions of Iran. Developed by integrating two internationally recognised tools – the NEMS-S^(24)^ and the INFORMAS Retail Module^(22)^ – the protocol was carefully designed to enable a thorough and multifaceted assessment of food environments. It was contextually tailored to align with the cultural, economic and nutritional characteristics specific to Iran’s urban areas.

The tool enables direct and structured observation of various types of food retail outlets, covering a spectrum of retail formats, including kiosks, small- and medium-sized food retailers (comparable to convenience stores) and large food retailers (comparable to grocery stores). The key dimensions assessed by this tool represent a synthesis of indicators from both the NEMS-S and INFORMAS instruments. These include the evaluation of product availability, prominence, quality, pricing and price comparison, as well as advertising and marketing strategies for individual food items and food groups. This combined method allows for a thorough, multifaceted assessment of the retail food environment and offers a practical framework for its monitoring, evaluation and comparison – supporting policy development and the design of targeted interventions to enhance the food environment.

The study applied Beaton *et al*.’s (2000) framework for translating and culturally adapting self-report measures^(30)^, involving the following steps to adapt this tool to the Iranian context:

1. **Translation.** First, the Retailer module from the INFORMAS website and the NEMS-S tool were downloaded. These tools were then translated from English to Farsi by two independent translators, both of whom were fluent in English and native speakers of Farsi. To ensure the accuracy and relevance of the translations, one translator possessed a comprehensive understanding of the study’s subject matter, while the other translator did not have this specialised knowledge. This approach aimed to balance technical accuracy with general comprehensibility.
2. **Synthesis.** At this stage, the two translators collaborated with the primary researcher, who acted as an evaluator, to merge the translation results. They meticulously compared the translations, identifying and addressing any discrepancies or differences. The resolution of these inconsistencies was achieved through consensus among all three participants, ensuring that the final translation was both accurate and coherent. This collaborative effort ensured the highest quality and reliability of the translated materials.
3. **Back-translation.** Two additional translators were hired to independently re-translate the protocols from Farsi back into English. Both of these translators were native English speakers fluent in Farsi, and neither had prior knowledge of the concepts within the protocol, nor were their backgrounds related to the study’s subject matter. This step aimed to ensure the accuracy and precision of the initial translations of the INFORMAS module and the NEMS-S tool. Once this re-translation process was complete, the initial draft of the protocol was presented to a panel of experts for further evaluation and validation.
4. **Expert committee review.** After the initial translation, the protocol was adapted to the Iranian context by removing irrelevant food items and adjusting others to reflect local products. For instance, ready-to-eat foods and dairy products were revised through field research to match those available in Iran. An initial invitation was sent via email to national specialists with relevant knowledge and research experience in nutrition and public health, expertise in evaluating retail food environments and familiarity with standard assessment tools. They were asked to confirm their willingness to participate in the expert panel. Ultimately, eleven experts agreed to take part. The updated protocol was then reviewed by this panel, which consisted of seven nutrition specialists, two school health experts and two environmental health specialists. Both the Persian and English versions of the protocol were provided for their evaluation. Using the Lawshe scale, validity indices were calculated based on their feedback, ensuring the protocol’s accuracy and relevance^(31)^.
5. **Pretesting.** This pilot study was conducted exclusively to evaluate the reliability of the assessment tool; therefore, the data collected during this phase were not used for environmental analysis but rather to determine inter-rater consistency. A clustered random sampling method was used for the pilot study. Based on data from the Deputy of Planning and Budget of Shiraz Municipality^(32)^, the city was divided into three areas representing high, middle and low socio-economic status. In each area, three stores were randomly selected across different retail types, including kiosks, small- and medium-sized food retailers and large food outlets, yielding a total of nine stores for the pilot phase. All necessary permissions were obtained in advance, and informed consent was secured from store managers.
6. **Submission and appraisal of all written reports.** After consulting with experts and conducting pretesting, we finalised the protocol by calculating its validity and reliability indices.

### Validity and reliability of indicators

The protocol was assessed based on two key dimensions: content validity and face validity. To evaluate content validity, we calculated two indices: the content validity ratio (CVR) and the content validity index (CVI). For this purpose, we developed a content validity assessment form that included all protocol items. Each item was rated by the expert panel on a five-point scale, ranging from 1 (very low relevance) to 5 (very high relevance), to determine its necessity and appropriateness. Experts were also invited to provide recommendations for refinement. The completed forms were collected via email, and the CVR was computed using the following equation:

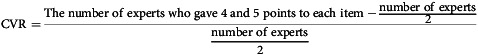




CVR values were evaluated against the minimum acceptable threshold established by Lawshe, which varies according to the number of experts on the panel^(31)^. Given that the expert panel consisted of eleven members, the threshold for the CVR was set at 0·59. To validate each item’s content, its CVR had to exceed this threshold^(31)^.

To determine the CVI, the survey questions were assessed on a four-point scale, where scores ranged from 1 to 4, evaluating each item’s simplicity, clarity and relevance. Here, relevance refers to the extent to which an item aligns with the objectives of the tool and the theoretical constructs it aims to measure. This scale provided a structured approach to rating how well each question met these criteria. The final CVI score was then calculated using the following formula:

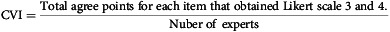




The CVI for each item was determined by averaging the scores for all three criteria: simplicity, clarity and relevance. Based on Lynn, a CVI value exceeding 0·78 was deemed acceptable^(33)^. Items with CVI values below 0·70 were excluded from the protocol, while those scoring between 0·70 and 0·78 were revised to improve their validity.

Face validity was evaluated by calculating the item impact score (IIS) using the following equation^(34)^:

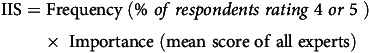




Experts were asked to assess each item based on its significance using a five-point Likert scale, where 1 indicated ‘very low’ importance, 2 ‘low’, 3 ‘moderate’, 4 ‘high’ and 5 ‘very high’. To gauge the importance of each item, we calculated two metrics: the frequency percentage of experts who rated the item as either 4 or 5 and the average score given by all experts. Items with an impact score below 1·5 were considered inadequate and subsequently removed from the tool to ensure that only the most significant items were retained^(34)^.

Reliability: To assess the reliability of the protocol, two researchers independently applied it across a diverse array of retail environments, including kiosks, small- and medium-sized food retailers and large food retailers. Inter-rater reliability was evaluated using both the intraclass correlation coefficient (ICC) and the percentage of agreement between the researchers. The ICC was used to measure the consistency of ratings between the two researchers, assessing how similarly they scored the same variables, such as each market’s rating. Additionally, the percentage of agreement was calculated to quantify the degree to which their assessments were aligned. An ICC value below 0·4 indicated poor inter-rater reliability, while a value above 0·75 indicated excellent inter-rater reliability. This approach ensured a thorough evaluation of the protocol’s reliability across different raters^(35)^. The agreement percentage was computed as follows:

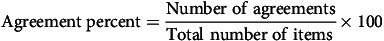




The acceptable limit of the agreement was considered higher than 90 %.

### Statistical analysis

Statistical analyses for CVR, CVI, IIS and agreement percentage were conducted in Excel 2016, while ICC for inter-rater reliability was computed using SPSS version 26.

## Results

The following section summarises the finalised measurement protocol and scoring system developed based on the methods described earlier, followed by the results of its validity and reliability assessments.

### Design

This protocol was designed to assess the status of the food environment across various types of food retailers, including kiosks, small- and medium-sized food retailers and large food retailers.

In the initial phase, food items were categorised based on the NEMS-S structure^(24)^. These categories were subsequently updated and localised using Iran’s Food-Based Dietary Guidelines^(36)^ and expert consultation, with careful consideration of local cultural norms, dietary patterns and food availability. The aim was to identify commonly consumed items and group them appropriately to reflect Iranian dietary culture. Ultimately, fourteen main food categories were defined (as presented in Table 1). Within each category, items were classified as either ‘healthy’ or ‘unhealthy’ based on national and regional criteria, including the WHO Eastern Mediterranean Region nutrient profiling model^(37)^; Iran’s national standards for sugar, salt and fat content^(38)^; the list of health-threatening products referenced in Article 37 of the Iran’s Fifth Development Plan^(39)^; and expert consultation. To enhance usability, in cases where classification was unclear due to variations in brand or packaging, researchers were instructed to rely on direct observation and food labelling (such as traffic light labelling) to make final determinations.

**Table 1.** Classification of food groups in the food environment analysis protocol: distinguishing between healthy and unhealthy options

Food groups	Healthy options	Unhealthy/less healthy options
Dairy products	Skim, low-fat or low-salt dairy products	Sweet or high-fat dairy products
Fruits	Fresh fruits and frozen fruits	Jam, fruit leather or dried fruits with added high sugar or salt, compote
Vegetables	Fresh or frozen vegetables	Salty canned vegetables and fried vegetables
Meat	White meat	High fat red meat
Hot dogs and sausages	Healthy vegan sausages	Unhealthy vegan sausages with high level of fat or salt, regular hot dogs and sausages
Ready-to-eat foods	Ready-to-eat foods with less than 10 g of fat/sugar per serving	Ready-to-eat foods with more than 10 g of fat/sugar per serving
Cakes, biscuits and cookies	Items containing bran or made from whole wheat flour or with reduced sugar	Regular items (made from refined wheat flour)
Beverages	Water and juice with more than 25 % natural juice	Soda, instant and sweetened coffee, diet soda, energy drinks, sports drinks and juice with less than 25 % natural juice
Bread and bakery products	All kinds of bread made from whole-grain flour or multi-grain flour	All kinds of bread made from refined flour or added sugar and/or high salt content
Chips and puffs	–	All kinds of chips and puffs, popcorn and tortilla chips
Breakfast cereals	Breakfast cereals with reduced sugar or fat or plain breakfast cereals	Flavoured breakfast cereal
Sweets	–	Candies, cocoa and chocolate, jelly, chewing gum and gumdrop
Rice, pasta and beans	Rice, pasta and different kinds of beans	–
Simple sugars	–	Sugar, honey and other kinds of simple sugars

Building on this foundational classification, a thorough and systematic approach was applied to evaluate the availability, prominence, quality, pricing and advertising of all listed food items.

Shelf space, a critical criterion for evaluating availability, was measured by length, depth and shelf count in accordance with INFORMAS retail module guidelines. The presence of healthy and unhealthy items across designated food groups was documented accordingly.

Prominence was assessed based on item placement and visibility within the store. Items were categorised as having high, moderate or low prominence depending on their location, with key areas such as checkout zones or Endcap A (shelf ends facing the front of the store or the main customer pathway) considered highly prominent. Shelf height was also recorded to assess visibility and accessibility, particularly for children.

Product quality, limited to fruits and vegetables, was assessed based on researcher observations of freshness and visual appeal, using scoring methods similar to the NEMS-S checklist.

In terms of pricing, product costs were recorded based on weight or volume. For each product, both the highest and lowest available prices were documented. Price data were primarily obtained from product labels; when unavailable, store personnel were consulted. Temporary or discounted (sale) prices were excluded unless they were the only listed price. This approach supports a more accurate evaluation of price disparities between healthy and unhealthy food categories.

Food advertisements were assessed both inside and outside the stores using a structured framework that recorded the size, content, use of characters and promotional techniques associated with each advertisement.

By integrating these dimensions, the protocol provides a multidimensional analysis of the retail food environment, following the INFORMAS and NEMS-S frameworks. It enables a comprehensive assessment of food item availability, prominence, price, quality and advertising within retail environments, offering valuable insights for monitoring and improving the food environment. Detailed scoring tools and evaluation criteria are provided in the Supplemental File, with a summary of dimensions and indicators presented in Table 2.

**Table 2.** Dimensions and components of the food environment analysis protocol for food retail stores: definitions and measures based on the INFORMAS and NEMS-S guidelines

Domain	Description	Assessment measures
Product availability	Assesses the presence of different food categories	Shelf space
Product prominence	Shelf placement in the store	Product shelf space based on its positioning within the store
Pricing	Price comparison of healthy *v*. unhealthy foods	Price comparison of healthy *v*. unhealthy foods
Food promotions	Evaluates the frequency and strategies of promotions inside and outside the store	Promotions frequency and strategies used in-store and externally
Product quality	Physical quality of products (exclusive to the fruits and vegetables group)	Freshness, appearance and spoilage level

### Scoring

The scoring system has been revised from the NEMS-S scoring system to accommodate new food categories and items, as well as updated methods for assessing specific dimensions. Stores can now be compared based on key criteria outlined in the guidelines, including availability, price, quality, prominence and the quantity and proportion of advertisements for healthy and unhealthy food items.

This system independently evaluates food retailers based solely on three dimensions: availability, price and quality of food items. The remaining two dimensions assessed in this protocol – product prominence and advertising – are presented only through descriptive reports and are not included in the final scoring system.

The maximum possible score for each quantitative dimension is as follows: availability (0–29), price (0–22) and food quality (0–6). These three scores can be combined to provide a comprehensive comparison across different retail stores. The highest achievable score is 57, with higher scores indicating a healthier food environment and lower scores reflecting a less healthy environment.

In the prominence domain, store areas are classified into high, medium and low prominence based on product placement. Within each category, the availability ratio of healthy to unhealthy products is determined and analysed across different levels of exposure. This method allows for a comprehensive assessment of whether healthier products are positioned more or less prominently than unhealthy ones, providing insight into the extent to which store layouts support healthier choices.

Similarly, advertising is assessed by evaluating the number of healthy and unhealthy ads both inside and outside the store:

1. In-store Advertising: This evaluation focuses on the promotion of healthy *v*. unhealthy products, based on the WHO Eastern Mediterranean Region nutrient profiling model^(37)^ as well as the list of health-threatening products referenced in Article 37 of Iran’s Fifth Development Plan^(39)^. Ads are categorised as healthy or unhealthy based on these criteria.
2. Advertising Outside the Store: This follows the INFORMAS advertising guidelines, which classify food products as healthy or unhealthy^(40)^. Advertisements are assessed accordingly based on the WHO Eastern Mediterranean Region nutrient profiling model^(37)^ as well as the list of health-threatening products referenced in Article 37 of Iran’s Fifth Development Plan^(39)^.

The number of healthy and unhealthy advertisements, both inside and outside the store, is reported separately and compared across stores to assess overall marketing strategies. Detailed scoring and comparison instructions are provided in Supplemental 3.

### Validity and reliability

Table 3 presents the results of the content and face validity assessments for the food groups. The CVR and CVI values were high, ranging from 0·64 to 1 and 0·78 to 1, respectively. Face validity, calculated using the IIS, was also high. Items that did not meet the specified cut-offs were removed from the final style sheet. After eliminating these items, two researchers reviewed the protocol to evaluate its reliability, resulting in a total agreement percentage of 93·77 %.

**Table 3.** Content validity and face validity of food groups in the food environment analysis protocol for retail stores: assessment based on CVR, CVI and IIS

Food groups	CVR^*^	CVI^†^	IIS^‡^
Dairy products	1	1	4·64
Fruits	0·66	0·78	2·84
Vegetables	0·82	0·96	3·97
Meat	0·64	0·78	3·35
Hot dogs and sausages	0·8	1	4·14
Ready-to-eat foods	0·82	0·85	4·05
Cakes, biscuits and cookies	0·78	0·81	4·05
Beverages	1	0·96	4·64
Bread and bakery products	0·82	1	4·13
Chips and puffs	0·8	0·89	4·05
Breakfast cereals	0·82	0·96	3·88
Sweets (e.g. candy, jelly, gums, etc.)	1	1	4·75
Rice, pasta and beans	0·82	0·96	4·14
Simple sugars	0·8	1	4·83

* Content validity ratio.

† Content validity index.

‡ Item Impact Score.

Table 4 summarises the findings for inter-rater reliability, using the ICC and agreement percentage for all food categories. The ICC and agreement percentage values for inter-rater reliability were consistently very high, ranging from 0·89 to 1 and 85 to 100 %, respectively.

**Table 4.** Inter-rater reliability for the food environment analysis protocol

Food groups	Inter-rater reliability	
	Intraclass correlation coefficient (ICC)	Agreement percent
Dairy	0·93	92·4 %
Fruits	1	100 %
Vegetables	0·96	95·2 %
Meat	0·89	85 %
Hot dogs and sausages	0·98	97·3 %
Ready-to-eat foods	0·91	89·4 %
Cakes, biscuits and cookies	0·98	97·6 %
Beverages	0·94	94 %
Bread and bakery products	1	100 %
Chips and puffs	0·94	93·5 %
Breakfast cereals	1	100 %
Sweets	0·89	87·9 %
Rice, pasta and beans	0·89	87·1 %
Simple sugars	0·94	93·4 %

## Discussion

This study adapted and validated a food environment analysis protocol for Iranian retail stores by integrating NEMS-S^(24)^ with the INFORMAS Retail Module^(22)^. Our tool combines rigorous psychometric criteria with practical, culturally relevant indicators to assess availability, quality, pricing, prominence and promotion of healthy *v*. unhealthy foods.

In recent years, various tools have been developed internationally to assess the food environment in retail settings, each with its own advantages and limitations. The SNAP-Ed Store Survey evaluates access to and affordability of healthy food items using nutrition coupons, offering a policy-oriented approach to promoting healthy eating among low-income groups^(41)^. However, it is tailored to the US context and may not translate easily to countries with different retail structures and food cultures. The Healthy Food Availability Index measures the availability and diversity of healthy food groups such as fruits, vegetables and low-fat products^(42)^ but overlooks critical elements like product placement, in-store advertising, packaging and pricing – factors that heavily influence consumer choices. The Grocery Store Quality Index examines overall store quality based on healthy food availability^(43)^ and yet fails to capture how unhealthy items are marketed. The CORE checklist adds pricing and layout indicators^(44)^ but can be time-consuming to implement and requires specialised training. Many of these tools also lack flexibility for culturally specific or local products, reducing their effectiveness in diverse or low-resource settings.

Overall, while these instruments capture key aspects of the retail food environment and permit cross-store comparisons, they face challenges including implementation complexity, extensive training needs, limited cultural adaptability and insufficient evaluation of marketing techniques and psychological influences. These gaps underscore the need for context-specific, user-friendly and comprehensive tools, particularly in low- and middle-income countries.

Among these tools and indices, although the NEMS-S instrument^(24)^ demonstrates considerable inter-rater reliability internationally (with kappa’s ranging from 0·83 to 1·00 and ICCs up to 0·98 across diverse settings), its regional adaptations introduce challenges in indicator selection and scoring methods^(24,29,45–47)^.

One of the primary challenges in assessing the food environment is classifying items as healthy or unhealthy. Many evaluation protocols rely on diverse criteria – such as the Mediterranean Food Pyramid, household budget analyses, national dietary intake surveys and processing-level nutrition guidelines^(24,29,47)^. Although these approaches offer diverse perspectives on food health, the simultaneous use of multiple classification systems creates complexity and increases the risk of inconsistency in evaluation. Furthermore, some of these indicators may not align with local dietary patterns and cultural preferences, potentially leading to a distorted view of the actual food environment. In the present study, to address this challenge, regional and national nutritional standards and indicators were employed. In cases where inconsistencies existed among these standards, the differences were discussed in expert panel meetings, and the classification of products was ultimately based on their nutritional traffic light labelling to ensure a more accurate categorisation of food items.

Another limitation lies in the NEMS-S tool used to assess food environments. Despite their strengths in evaluating availability, price and quality, different versions of the NEMS-S tool do not account for in-store and surrounding food advertising or the prominence of food placement. However, recent research highlights the significant influence of product marketing and display on consumer choices^(48,49)^.

Ultimately, by integrating key elements from internationally recognised tools and tailoring them to the local cultural, social and dietary context, we developed an expanded, flexible and context-sensitive approach to more accurately assess the food environment of stores; despite these methodological differences, our findings suggest that the adapted tool performs consistently across diverse retail settings and maintains its capacity to measure the same underlying constructs, thereby supporting its utility for future research and monitoring efforts aimed at promoting healthier food environments – particularly in middle-income countries with varied retail landscapes.

### Implications for policy and practice

This study, by validating a comprehensive and localised protocol for assessing the food environment in retail stores in Iran, has made an important contribution to improving policymaking and practical actions in public health. The developed tool, considering various aspects such as access, pricing, quality, product placement and food advertising, provides a multidimensional and realistic picture of the retail food environment.

Moreover, with its user-friendly design, the tool can also be utilised by store managers to assess the store’s status and enhance the availability of healthy foods. This dual application strengthens the tool’s significance not only in the field of research but also in promoting practical improvements in retail food environments at the national level.

By incorporating these diverse dimensions and addressing the local context, the tool offers a more nuanced and culturally relevant evaluation of food environments in Iran. It also provides valuable data that can be used to inform interventions aimed at improving food access and promoting healthier dietary habits among the population. As such, it holds promise for long-term positive changes in both research and public health initiatives across the country.

### Strengths and limitations

This study represents the first adaptation and validation of a food environment assessment tool for Iranian stores, with high inter-rater reliability confirming its robustness and ease of use. However, limitations exist. The protocol was tested in a single city (Shiraz), necessitating further validation across different regions of Iran. Additionally, seasonal and geographic variations in food availability could affect the classification of food items, requiring ongoing adjustments to ensure accuracy.

### Conclusion

In conclusion, this study successfully adapted and validated a food environment assessment tool for retail stores in Iran, combining international standards with local cultural and dietary contexts. By incorporating key aspects such as food availability, pricing, quality, placement and marketing, the tool offers a comprehensive and context-sensitive approach for evaluating retail food environments. Its user-friendly design allows for both research and practical applications, enabling store managers to assess and improve the availability of healthy foods in their establishments.

## Supporting information

Rostami et al. supplementary material Rostami et al. supplementary material

## Supplementary material

For supplementary material accompanying this paper, visit https://doi.org/10.1017/S1368980025100773.
