# Supplementary material for: Development and validation of a food retail environment analysis protocol in Iran
Source: Public Health Nutr. 2025 Aug 5;28(1):e132. doi: 10.1017/S1368980025100773 (PMC12465068; doi:10.1017/S1368980025100773)
Supplement: Rostami et al. supplementary material [file S1368980025100773sup001.docx]

**The checklist of Retail Food Environment Analysis**

**Assessor's code:** .......... **Store name:** ………. **Store type:** Kiosk□ small supermarket□ Medium/Large Supermarket□ chain store□ **Date of Visit:** ...../ ...../ .......... **Start Time:** ..... : ..... **End Time:** ..... : ..... **Number of Cash Registers:** .............. **Number of Aisles:** .......... **Store Floor Area:** ......... **Neighborhood Name:** .............. **Consent Obtained from Owner/Manager:** yes□ no□

**Measure 1- Milk and dairy**

Are **milk and dairy** products available in this store? Yes□ No□

**Availability and prominence**

| **Food item** | **volume** | **Shelf location** | **Shelf height (Cm)** | **Shelf length**  **(Cm)** | **Shelf depth (Cm)** | **Number of shelves** | **Percentage availability of low-fat or semi-fat milk, yogurt, or cheese / low-salt buttermilk on the shelf** | **Percentage availability of full-fat dairy products / regular buttermilk on the shelf** | **Percentage availability of flavored dairy products on the shelves** | **Percentage availability of plant-based dairy products on the shelves** |
| --- | --- | --- | --- | --- | --- | --- | --- | --- | --- | --- |
| **Milk** | …… |  |  |  |  |  |  |  |  |  |
|  | …… |  |  |  |  |  |  |  |  |  |
|  | …… |  |  |  |  |  |  |  |  |  |
|  | …… |  |  |  |  |  |  |  |  |  |
| **Yogurt** | …… |  |  |  |  |  |  |  |  |  |
|  | …… |  |  |  |  |  |  |  |  |  |
|  | …… |  |  |  |  |  |  |  |  |  |
|  | …… |  |  |  |  |  |  |  |  |  |
| **Dough (Yoghurt drink)** | …… |  |  |  |  |  |  |  |  |  |
|  | …… |  |  |  |  |  |  |  |  |  |
|  | …… |  |  |  |  |  |  |  |  |  |
|  | …… |  |  |  |  |  |  |  |  |  |
| **Cheese** | …… |  |  |  |  |  |  |  |  |  |
|  | …… |  |  |  |  |  |  |  |  |  |
|  | …… |  |  |  |  |  |  |  |  |  |
|  | …… |  |  |  |  |  |  |  |  |  |

**Price**

| **Food item** | **Types** | **Characteristics:**  **Fat percentage / Salt percentage** | **Volume** | **Price** | **Details** |
| --- | --- | --- | --- | --- | --- |
| **Milk** | Healthy | Low-fat or semi-skimmed  / Plant-based without added sugar | .................... | Cheapest product: ………….………………....  Most expensive product: …….…………..….. |  |
|  | Unhealthy | High-fat/ flavored with added sugar | .................... | Cheapest product: ………….………………....  Most expensive product: …….…………..….. |  |
| **Yogurt** | Healthy | Low-fat or semi-skimmed | .................... | Cheapest product: ………….………………....  Most expensive product: …….…………..….. |  |
|  | Unhealthy | High-fat or creamy or added sugar | .................... | Cheapest product: ………….………………....  Most expensive product: …….…………..….. |  |
| **Dough (Yoghurt drink(** | Healthy | Low-salt | .................... | Cheapest product: ………….………………....  Most expensive product: …….…………..….. |  |
|  | Unhealthy | High-salt | .................... | Cheapest product: ………….………………....  Most expensive product: …….…………..….. |  |
| **Cheese** | Healthy | Low-fat or semi-skimmed, low salt | .................... | Cheapest product: ………….………………....  Most expensive product: …….…………..….. |  |
|  | Unhealthy | High-fat or creamy, High salt | .................... | Cheapest product: ………….………………....  Most expensive product: …….…………..….. |  |

**Measure 2- Fruits**

Are **fresh fruit** products available in this store? Yes□ No□

**Availability, prominence, and price**

| **Healthy options** | | | | | | | | | | | | |
| --- | --- | --- | --- | --- | --- | --- | --- | --- | --- | --- | --- | --- |
| **Food item** | **Type** | **Availability** | | **Shelf location** | **Shelf height (Cm)** | **Shelf length**  **(Cm)** | **Shelf depth (Cm)** | **Number of shelves** | **Quality** | | **Price per Kg/ unit** | **Details** |
|  |  | **Yes** | **No** |  |  |  |  |  | **High** | **Low** |  |  |
| **Banana** |  |  |  |  |  |  |  |  |  |  | Lowest-priced: …………  Highest-priced: ………… |  |
| **Apple** |  |  |  |  |  |  |  |  |  |  | Lowest-priced: …………  Highest-priced: ………… |  |
| **Orange** |  |  |  |  |  |  |  |  |  |  | Lowest-priced: …………  Highest-priced: ………… |  |
| **Grape** |  |  |  |  |  |  |  |  |  |  | Lowest-priced: …………  Highest-priced: ………… |  |
| **Melon** |  |  |  |  |  |  |  |  |  |  | Lowest-priced: …………  Highest-priced: ………… |  |
| **Peach** |  |  |  |  |  |  |  |  |  |  | Lowest-priced: …………  Highest-priced: ………… |  |
| **Strawberry** |  |  |  |  |  |  |  |  |  |  | Lowest-priced: …………  Highest-priced: ………… |  |
| **Watermelon** |  |  |  |  |  |  |  |  |  |  | Lowest-priced: …………  Highest-priced: ………… |  |
| **Plum** |  |  |  |  |  |  |  |  |  |  | Lowest-priced: …………  Highest-priced: ………… |  |
| **Nectarine** |  |  |  |  |  |  |  |  |  |  | Lowest-priced: …………  Highest-priced: ………… |  |
| **Mixed fruits** |  |  |  |  |  |  |  |  |  |  | Lowest-priced: …………  Highest-priced: ………… | Include: |
| **Frozen fruits** | ……... |  |  |  |  |  |  |  |  |  | Lowest-priced: …………  Highest-priced: ………… |  |
|  | ……... |  |  |  |  |  |  |  |  |  | Lowest-priced: …………  Highest-priced: ………… |  |
|  | ……... |  |  |  |  |  |  |  |  |  | Lowest-priced: …………  Highest-priced: ………… |  |
|  | ……... |  |  |  |  |  |  |  |  |  | Lowest-priced: …………  Highest-priced: ………… |  |
|  | ……... |  |  |  |  |  |  |  |  |  | Lowest-priced: …………  Highest-priced: ………… |  |
|  | ……... |  |  |  |  |  |  |  |  |  | Lowest-priced: …………  Highest-priced: ………… |  |
| **Other seasonal fruits**  **Details:** |  |  |  |  |  |  |  |  |  |  | Lowest-priced: …………  Highest-priced: ………… |  |

Are **processed fruit** products available in this store? Yes□ No□

**Availability, prominence, and price**

| **Unhealthy options** | | | | | | | | | | | |
| --- | --- | --- | --- | --- | --- | --- | --- | --- | --- | --- | --- |
| **Food item** | **Weight** | **Availability** | | **Shelf location** | **Shelf height (Cm)** | **Shelf length**  **(Cm)** | **Shelf depth (Cm)** | **Number of shelves** | **Price** | **Type** | **Details** |
|  |  | **Yes** | **No** |  |  |  |  |  |  |  |  |
| Jam | .......................... |  |  |  |  |  |  |  | Lowest-priced: …………….  Highest-priced:…………..… |  |  |
|  | .......................... |  |  |  |  |  |  |  | Lowest-priced: …………….  Highest-priced:…………..… |  |  |
|  | .......................... |  |  |  |  |  |  |  | Lowest-priced: …………….  Highest-priced:…………..… |  |  |
|  | .......................... |  |  |  |  |  |  |  | Lowest-priced: …………….  Highest-priced:…………..… |  |  |
|  | .......................... |  |  |  |  |  |  |  | Lowest-priced: …………….  Highest-priced:…………..… |  |  |
| Dried fruit with added salt or sugar | .......................... |  |  |  |  |  |  |  | Lowest-priced: …………….  Highest-priced:…………..… |  |  |
|  | .......................... |  |  |  |  |  |  |  | Lowest-priced: …………….  Highest-priced:…………..… |  |  |
|  | .......................... |  |  |  |  |  |  |  | Lowest-priced: …………….  Highest-priced:…………..… |  |  |
|  | .......................... |  |  |  |  |  |  |  | Lowest-priced: …………….  Highest-priced:…………..… |  |  |
|  | .......................... |  |  |  |  |  |  |  | Lowest-priced: …………….  Highest-priced:…………..… |  |  |
| Fruit compote | .......................... |  |  |  |  |  |  |  | Lowest-priced: …………….  Highest-priced:…………..… |  |  |
|  | .......................... |  |  |  |  |  |  |  | Lowest-priced: …………….  Highest-priced:…………..… |  |  |
|  | .......................... |  |  |  |  |  |  |  | Lowest-priced: …………….  Highest-priced:…………..… |  |  |
|  | .......................... |  |  |  |  |  |  |  | Lowest-priced: …………….  Highest-priced:…………..… |  |  |

**Measure 3- Vegetables**

Are **fresh vegetable** products available in this store? Yes□ No□

**Availability, prominence, and price**

| **Healthy options** | | | | | | | | | | | | | |
| --- | --- | --- | --- | --- | --- | --- | --- | --- | --- | --- | --- | --- | --- |
| **Food item** | **Type** | **Availability** | | **Shelf location** | **Shelf height (Cm)** | **Shelf length**  **(Cm)** | **Shelf depth (Cm)** | **Number of shelves** | **Quality** | | **Price per Kg/ unit** | **Details** |  |
|  |  | **Yes** | **No** |  |  |  |  |  | **High** | **Low** |  |  |  |
| Carrot |  |  |  |  |  |  |  |  |  |  | Lowest-priced: …...  Highest-price: ....… |  |  |
| Tomato |  |  |  |  |  |  |  |  |  |  | Lowest-priced: …...  Highest-price: ....… |  |  |
| bell pepper |  |  |  |  |  |  |  |  |  |  | Lowest-priced: …...  Highest-price: ....… |  |  |
| Broccoli |  |  |  |  |  |  |  |  |  |  | Lowest-priced: …...  Highest-price: ....… |  |  |
| Lettuce |  |  |  |  |  |  |  |  |  |  | Lowest-priced: …...  Highest-price: ....… |  |  |
| Corn |  |  |  |  |  |  |  |  |  |  | Lowest-priced: …...  Highest-price: ....… |  |  |
| Celery |  |  |  |  |  |  |  |  |  |  | Lowest-priced: …...  Highest-price: ....… |  |  |
| Cucumber |  |  |  |  |  |  |  |  |  |  | Lowest-priced: …...  Highest-price: ....… |  |  |
| Cabbage |  |  |  |  |  |  |  |  |  |  | Lowest-priced: …...  Highest-price: ....… |  |  |
| Cauliflower |  |  |  |  |  |  |  |  |  |  | Lowest-priced: …...  Highest-price: ....… |  |  |
| Potato |  |  |  |  |  |  |  |  |  |  | Lowest-priced: …...  Highest-price: ....… |  |  |
| Onion |  |  |  |  |  |  |  |  |  |  | Lowest-priced: …...  Highest-price: ....… |  |  |
| Eggplant |  |  |  |  |  |  |  |  |  |  | Lowest-priced: …...  Highest-price: ....… |  |  |
| Pumpkin |  |  |  |  |  |  |  |  |  |  | Lowest-priced: …...  Highest-price: ....… |  |  |
| pre-packaged vegetable mixes | .......................... |  |  |  |  |  |  |  |  |  | Lowest-priced: …...  Highest-price: ....… |  |  |
|  | .......................... |  |  |  |  |  |  |  |  |  | Lowest-priced: …...  Highest-price: ....… |  |  |
|  | .......................... |  |  |  |  |  |  |  |  |  | Lowest-priced: …...  Highest-price: ....… |  |  |
|  | .......................... |  |  |  |  |  |  |  |  |  | Lowest-priced: …...  Highest-price: ....… |  |  |
|  | .......................... |  |  |  |  |  |  |  |  |  | Lowest-priced: …...  Highest-price: ....… |  |  |
| Frozen vegetables | .......................... |  |  |  |  |  |  |  |  |  | Lowest-priced: …...  Highest-price: ....… |  |  |
|  | .......................... |  |  |  |  |  |  |  |  |  | Lowest-priced: …...  Highest-price: ....… |  |  |
|  | .......................... |  |  |  |  |  |  |  |  |  | Lowest-priced: …...  Highest-price: ....… |  |  |
|  | .......................... |  |  |  |  |  |  |  |  |  | Lowest-priced: …...  Highest-price: ....… |  |  |
|  | .......................... |  |  |  |  |  |  |  |  |  | Lowest-priced: …...  Highest-price: ....… |  |  |
| Other: |  |  |  |  |  |  |  |  |  |  | Lowest-priced: …...  Highest-price: ....… |  |  |

Are **processed vegetable** products available in this store? Yes□ No□

**Availability, prominence, and price**

| **Unhealthy options** | | | | | | | | | | | | | |
| --- | --- | --- | --- | --- | --- | --- | --- | --- | --- | --- | --- | --- | --- |
| **Food item** | **weight** | **Availability** | | **Shelf location** | **Shelf height (Cm)** | **Shelf length**  **(Cm)** | **Shelf depth (Cm)** | **Number of shelves** | **Quality** | | **Price** | **Type** | **Details** |
|  |  | **Yes** | **No** |  |  |  |  |  | **High** | **Low** |  |  |  |
| Canned vegetables | …….. |  |  |  |  |  |  |  |  |  | Lowest-priced: ......  Highest-price: …... |  |  |
|  | …….. |  |  |  |  |  |  |  |  |  | Lowest-priced: ......  Highest-price: …... |  |  |
|  | …….. |  |  |  |  |  |  |  |  |  | Lowest-priced: ......  Highest-price: …... |  |  |
|  | …….. |  |  |  |  |  |  |  |  |  | Lowest-priced: ......  Highest-price: …... |  |  |
| Fried vegetables | …….. |  |  |  |  |  |  |  |  |  | Lowest-priced: ......  Highest-price: …... |  |  |
|  | …….. |  |  |  |  |  |  |  |  |  | Lowest-priced: ......  Highest-price: …... |  |  |
|  | …….. |  |  |  |  |  |  |  |  |  | Lowest-priced: ......  Highest-price: …... |  |  |
|  | …….. |  |  |  |  |  |  |  |  |  | Lowest-priced: ......  Highest-price: …... |  |  |
|  | …….. |  |  |  |  |  |  |  |  |  | Lowest-priced: ......  Highest-price: …... |  |  |

**Measure 4- Meats**

Are **fresh meat** products available in this store? Yes□ No□

**Availability, prominence, and price**

| **Food item** | **Availability** | | **Shelf location** | **Shelf height (Cm)** | **Shelf length**  **(Cm)** | **Shelf depth (Cm)** | **Number of shelves** | **Price per Kg/ Package weight** | **Details** |
| --- | --- | --- | --- | --- | --- | --- | --- | --- | --- |
|  | **Yes** | **No** |  |  |  |  |  |  |  |
| **Healthy options (white meat)** | | | | | | | | | |
| Chicken meat |  |  |  |  |  |  |  | Lowest-priced: .……....  Highest-price: ……..… |  |
| Turkey or other poultry meat |  |  |  |  |  |  |  | Lowest-priced: .……....  Highest-price: ……..… |  |
| Fish |  |  |  |  |  |  |  | Lowest-priced: .……....  Highest-price: ……..… |  |
| Shrimp |  |  |  |  |  |  |  | Lowest-priced: .……....  Highest-price: ……..… |  |
| Eggs |  |  |  |  |  |  |  | Lowest-priced: .……....  Highest-price: ……..… |  |
| **Unhealthy options (red meat** **without fat trimming)** | | | | | | | | | |
| Regular beef |  |  |  |  |  |  |  | Lowest-priced: .……....  Highest-price: ……..… |  |
| Regular lamb |  |  |  |  |  |  |  | Lowest-priced: .……....  Highest-price: ……..… |  |
| Ground beef |  |  |  |  |  |  |  | Lowest-priced: .……....  Highest-price: ……..… |  |
| Mix ground meat |  |  |  |  |  |  |  | Lowest-priced: .……....  Highest-price: ……..… |  |

**Measure 5- Hot dog, sausage, and deli meat**

Are **hot dogs, sausage, and deli meat** products available in this store? Yes□ No□

**Availability, prominence, and price**

| **Food item** | **Type** | **Availability** | | **Shelf location** | **Shelf height (Cm)** | **Shelf length**  **(Cm)** | **Shelf depth (Cm)** | **Number of shelves** | **Price per Kg** | **Details** |  |
| --- | --- | --- | --- | --- | --- | --- | --- | --- | --- | --- | --- |
|  |  | **Yes** | **No** |  |  |  |  |  |  |  |  |
| **Healthy options** | | | | | | | | | | |  |
| Vegetarian healthy Product |  |  |  |  |  |  |  |  | Lowest-priced: .……....  Highest-price: ……..… |  |  |
|  |  |  |  |  |  |  |  |  | Lowest-priced: .……....  Highest-price: ……..… |  |  |
|  |  |  |  |  |  |  |  |  | Lowest-priced: .……....  Highest-price: ……..… |  |  |
| **Unhealthy options** | | | | | | | | | | | |
| Vegetarian unhealthy product |  |  |  |  |  |  |  |  | Lowest-priced: .……....  Highest-price: ……..… |  |  |
|  |  |  |  |  |  |  |  |  | Lowest-priced: .……....  Highest-price: ……..… |  |  |
|  |  |  |  |  |  |  |  |  | Lowest-priced: .……....  Highest-price: ……..… |  |  |
| Regular Hot dog, sausage, or deli meat |  |  |  |  |  |  |  |  | Lowest-priced: .……....  Highest-price: ……..… |  |  |
|  |  |  |  |  |  |  |  |  | Lowest-priced: .……....  Highest-price: ……..… |  |  |
|  |  |  |  |  |  |  |  |  | Lowest-priced: .……....  Highest-price: ……..… |  |  |

**Measure 6- Ready-to-eat food**

Are **ready-to-eat food** products available in this store? Yes□ No□

**Availability, prominence, and price**

| **Food item** | **Type** | **Availability** | | **Shelf location** | **Shelf height (Cm)** | **Shelf length**  **(Cm)** | **Shelf depth (Cm)** | **Number of shelves** | **Price** | **Weight** |  |
| --- | --- | --- | --- | --- | --- | --- | --- | --- | --- | --- | --- |
|  |  | Yes | No |  |  |  |  |  |  |  |  |
| **Healthy options** | | | | | | | | | | | |
| Ready-to-eat foods with reduced fat or with fat content less than or equal to 10 grams per serving: including various types of ready-made stews, canned tuna, various canned foods, ready-made sandwiches, hamburgers, falafel, nuggets, lasagna, pizza, fried fillet, kebab rolls, and more |  |  |  |  |  |  |  |  | Lowest-priced: .……....  Highest-price: ……..… |  |  |
|  |  |  |  |  |  |  |  |  | Lowest-priced: .……....  Highest-price: ……..… |  |  |
|  |  |  |  |  |  |  |  |  | Lowest-priced: .……....  Highest-price: ……..… |  |  |
|  |  |  |  |  |  |  |  |  | Lowest-priced: .……....  Highest-price: ……..… |  |  |
|  |  |  |  |  |  |  |  |  | Lowest-priced: .……....  Highest-price: ……..… |  |  |
|  |  |  |  |  |  |  |  |  | Lowest-priced: .……....  Highest-price: ……..… |  |  |
|  |  |  |  |  |  |  |  |  | Lowest-priced: .……....  Highest-price: ……..… |  |  |
| **Unhealthy options** | | | | | | | | | | |  |
| Regular ready-to-eat foods with more than 10 grams of fat per serving: including various types of ready-made stews, canned tuna, various canned foods, ready-made sandwiches, hamburgers, falafel, nuggets, lasagna, pizza, fried fillet, kebab rolls, and more. |  |  |  |  |  |  |  |  | Lowest-priced: .……....  Highest-price: ……..… |  |  |
|  |  |  |  |  |  |  |  |  | Lowest-priced: .……....  Highest-price: ……..… |  |  |
|  |  |  |  |  |  |  |  |  | Lowest-priced: .……....  Highest-price: ……..… |  |  |
|  |  |  |  |  |  |  |  |  | Lowest-priced: .……....  Highest-price: ……..… |  |  |
|  |  |  |  |  |  |  |  |  | Lowest-priced: .……....  Highest-price: ……..… |  |  |
|  |  |  |  |  |  |  |  |  | Lowest-priced: .……....  Highest-price: ……..… |  |  |
|  |  |  |  |  |  |  |  |  | Lowest-priced: .……....  Highest-price: ……..… |  |  |

**Measure 7- Cake, cookie, and biscuit**

Are **cake, cookie, and biscuit** products available in this store? Yes□ No□

**Availability, prominence, and price**

| **Food item** | **Availability** | | **Shelf location** | **Shelf height (Cm)** | **Shelf length**  **(Cm)** | **Shelf depth (Cm)** | **Number of shelves** | **Price** | **weight** |
| --- | --- | --- | --- | --- | --- | --- | --- | --- | --- |
|  | **Yes** | **No** |  |  |  |  |  |  |  |
| **Healthy options (foods containing bran, made from whole wheat flour, or foods with reduced sugar)** | | | | | | | | | |
| Plain bran cake without cream or chocolate |  |  |  |  |  |  |  | Lowest-priced: .…....  Highest-price: …..… |  |
| Plain bran biscuit without cream or chocolate |  |  |  |  |  |  |  | Lowest-priced: .…....  Highest-price: …..… |  |
| Plain bran cookie without cream or chocolate |  |  |  |  |  |  |  | Lowest-priced: .…....  Highest-price: …..… |  |
| Plain rice cake |  |  |  |  |  |  |  | Lowest-priced: .…....  Highest-price: …..… |  |
| **Alternative options** | | | | | | | | | |
| Plain diet biscuit (sugar-free or with reduced sugar) |  |  |  |  |  |  |  | Lowest-priced: .…....  Highest-price: …..… |  |
| Plain diet cake (sugar-free or with reduced sugar) |  |  |  |  |  |  |  | Lowest-priced: .…....  Highest-price: …..… |  |
| Plain diet cookie (sugar-free or with reduced sugar) |  |  |  |  |  |  |  | Lowest-priced: .…....  Highest-price: …..… |  |
| **Unhealthy options (foods made from refined wheat flour).** | | | | | | | | | |
| Plain biscuit |  |  |  |  |  |  |  | Lowest-priced: .…....  Highest-price: …..… |  |
| Flavored biscuit |  |  |  |  |  |  |  | Lowest-priced: .…....  Highest-price: …..… |  |
| Plain cake |  |  |  |  |  |  |  | Lowest-priced: .…....  Highest-price: …..… |  |
| Flavored cake |  |  |  |  |  |  |  | Lowest-priced: .…....  Highest-price: …..… |  |
| Plain cookie |  |  |  |  |  |  |  | Lowest-priced: .…....  Highest-price: …..… |  |
| Flavored cookie |  |  |  |  |  |  |  | Lowest-priced: .…....  Highest-price: …..… |  |
| Other types of cake, biscuit, and cookie |  |  |  |  |  |  |  | Lowest-priced: .…....  Highest-price: …..… |  |

**Measure 8- Beverages**

Are **beverage** products available in this store? Yes□ No□

**Availability, prominence, and price- Part 1**

| **Food item** | **Volume** | **Availability** | | **Shelf location** | **Shelf height (Cm)** | **Shelf length**  **(Cm)** | **Shelf depth (Cm)** | **Number of shelves** | **Price** | **Details** |
| --- | --- | --- | --- | --- | --- | --- | --- | --- | --- | --- |
|  |  | **Yes** | **No** |  |  |  |  |  |  |  |
| **Healthy options** | | | | | | | | | | |
| Water | ............ |  |  |  |  |  |  |  | Lowest-priced: .……………......  Highest-price: ……………....… |  |
|  | ............ |  |  |  |  |  |  |  | Lowest-priced: .……………......  Highest-price: ……………....… |  |
|  | ............ |  |  |  |  |  |  |  | Lowest-priced: .……………......  Highest-price: ……………....… |  |
|  | ............ |  |  |  |  |  |  |  | Lowest-priced: .……………......  Highest-price: ……………....… |  |
| **Unhealthy options** | | | | | | | | | | |
| Diet soda | ............ |  |  |  |  |  |  |  | Lowest-priced: .……………......  Highest-price: ……………....… |  |
|  | ............ |  |  |  |  |  |  |  | Lowest-priced: .……………......  Highest-price: ……………....… |  |
|  | ............ |  |  |  |  |  |  |  | Lowest-priced: .……………......  Highest-price: ……………....… |  |
|  | ............ |  |  |  |  |  |  |  | Lowest-priced: .……………......  Highest-price: ……………....… |  |
| Regular soda | ............ |  |  |  |  |  |  |  | Lowest-priced: .……………......  Highest-price: ……………....… |  |
|  | ............ |  |  |  |  |  |  |  | Lowest-priced: .……………......  Highest-price: ……………....… |  |
|  | ............ |  |  |  |  |  |  |  | Lowest-priced: .……………......  Highest-price: ……………....… |  |
|  | ............ |  |  |  |  |  |  |  | Lowest-priced: .……………......  Highest-price: ……………....… |  |
| Instant and sweetened coffee | ............ |  |  |  |  |  |  |  | Lowest-priced: .……………......  Highest-price: ……………....… |  |
|  | ............ |  |  |  |  |  |  |  | Lowest-priced: .……………......  Highest-price: ……………....… |  |
|  | ............ |  |  |  |  |  |  |  | Lowest-priced: .……………......  Highest-price: ……………....… |  |
| Energy drink | ............ |  |  |  |  |  |  |  | Lowest-priced: .……………......  Highest-price: ……………....… |  |
|  | ............ |  |  |  |  |  |  |  | Lowest-priced: .……………......  Highest-price: ……………....… |  |
|  | ............ |  |  |  |  |  |  |  | Lowest-priced: .……………......  Highest-price: ……………....… |  |
|  | ............ |  |  |  |  |  |  |  | Lowest-priced: .……………......  Highest-price: ……………....… |  |
| Sports drink | ............ |  |  |  |  |  |  |  | Lowest-priced: .……………......  Highest-price: ……………....… |  |
|  | ............ |  |  |  |  |  |  |  | Lowest-priced: .……………......  Highest-price: ……………....… |  |
|  | ............ |  |  |  |  |  |  |  | Lowest-priced: .……………......  Highest-price: ……………....… |  |
|  | ............ |  |  |  |  |  |  |  | Lowest-priced: .……………......  Highest-price: ……………....… |  |

**Availability, prominence, and price- Part 2**

| **Healthy options** | | | | | | | | | | |
| --- | --- | --- | --- | --- | --- | --- | --- | --- | --- | --- |
| Fruit juice with more than 25% natural content without added sugar | ............ |  |  |  |  |  |  |  | Lowest-priced: .……………......  Highest-price: ……………....… |  |
|  | ............ |  |  |  |  |  |  |  | Lowest-priced: .……………......  Highest-price: ……………....… |  |
|  | ............ |  |  |  |  |  |  |  | Lowest-priced: .……………......  Highest-price: ……………....… |  |
| **Unhealthy options** | | | | | | | | | | |
| Beverages containing fruit juice (equal to or less than 25% natural content) | ............ |  |  |  |  |  |  |  | Lowest-priced: .……………......  Highest-price: ……………....… |  |
|  | ............ |  |  |  |  |  |  |  | Lowest-priced: .……………......  Highest-price: ……………....… |  |
|  | ............ |  |  |  |  |  |  |  | Lowest-priced: .……………......  Highest-price: ……………....… |  |

**Measure 9- Bread and bakery products**

Are **bread and bakery products** available in this store? Yes□ No□

**Availability, prominence, and price**

| **Food item** | **Availability** | | **Shelf location** | **Shelf height (Cm)** | **Shelf length**  **(Cm)** | **Shelf depth (Cm)** | **Number of shelves** | **Weight** | **Price** | **Details** |  |
| --- | --- | --- | --- | --- | --- | --- | --- | --- | --- | --- | --- |
|  | **Yes** | **No** |  |  |  |  |  |  |  |  |  |
| **Healthy options (varieties of bread made from whole-grain or multi-grain flour, or breads without added sugar and with reduced salt)** | | | | | | | | | | | |
| ............................................. |  |  |  |  |  |  |  |  | Lowest-priced: .……........  Highest-price:……....… |  |  |
| ............................................. |  |  |  |  |  |  |  |  | Lowest-priced: .……........  Highest-price:……....… |  |  |
| ............................................. |  |  |  |  |  |  |  |  | Lowest-priced: .……........  Highest-price:……....… |  |  |
| ............................................. |  |  |  |  |  |  |  |  | Lowest-priced: .……........  Highest-price:……....… |  |  |
| **Unhealthy options (varieties of bread made from refined flour, with added sugar and/or high salt content)** | | | | | | | | | | | |
| ............................................. |  |  |  |  | 0 |  |  |  | Lowest-priced: .……........  Highest-price:……....… |  |  |
|  |  |  |  |  |  |  |  |  |  |  |  |
| ............................................. |  |  |  |  |  |  |  |  | Lowest-priced: .……........  Highest-price:……....… |  |  |
| ............................................. |  |  |  |  |  |  |  |  | Lowest-priced: .……........  Highest-price:……....… |  |  |
| ............................................. |  |  |  |  |  |  |  |  | Lowest-priced: .……........  Highest-price:……....… |  |  |

**Measure 10- Chips and cheese puffs**

Are **chips and cheese puffs** products available in this store? Yes□ No□

**Availability, prominence, and price**

| **Food item** | **weight** | **Availability** | | **Shelf location** | **Shelf height (Cm)** | **Shelf length**  **(Cm)** | **Shelf depth (Cm)** | **Number of shelves** | **Price** | **Type** |  |
| --- | --- | --- | --- | --- | --- | --- | --- | --- | --- | --- | --- |
|  |  | **Yes** | **No** |  |  |  |  |  |  |  |  |
| **Healthy options** | | | | | | | | | | | |
| Lowest fat & salt variant***** |  |  |  |  |  |  |  |  | Lowest-priced: ………........  Highest-price:…………...… |  |  |
|  |  |  |  |  |  |  |  |  | Lowest-priced: ………........  Highest-price:…………...… |  |  |
|  |  |  |  |  |  |  |  |  | Lowest-priced: ………........  Highest-price:…………...… |  |  |
|  |  |  |  |  |  |  |  |  | Lowest-priced: ………........  Highest-price:…………...… |  |  |
| **Unhealthy options** | | | | | | | | | | |  |
| Potato chips | ..................... |  |  |  |  |  |  |  | Lowest-priced: ………........  Highest-price:…………...… |  |  |
|  | ..................... |  |  |  |  |  |  |  | Lowest-priced: ………........  Highest-price:…………...… |  |  |
|  | ..................... |  |  |  |  |  |  |  | Lowest-priced: ………........  Highest-price:…………...… |  |  |
|  | ..................... |  |  |  |  |  |  |  | Lowest-priced: ………........  Highest-price:…………...… |  |  |
| cheese puffs | ..................... |  |  |  |  |  |  |  | Lowest-priced: ………........  Highest-price:…………...… |  |  |
|  | ..................... |  |  |  |  |  |  |  | Lowest-priced: ………........  Highest-price:…………...… |  |  |
|  | ..................... |  |  |  |  |  |  |  | Lowest-priced: ………........  Highest-price:…………...… |  |  |
|  | ..................... |  |  |  |  |  |  |  | Lowest-priced: ………........  Highest-price:…………...… |  |  |
| popcorn | ..................... |  |  |  |  |  |  |  | Lowest-priced: ………........  Highest-price:…………...… |  |  |
|  | ..................... |  |  |  |  |  |  |  | Lowest-priced: ………........  Highest-price:…………...… |  |  |
|  | ..................... |  |  |  |  |  |  |  | Lowest-priced: ………........  Highest-price:…………...… |  |  |
|  | ..................... |  |  |  |  |  |  |  | Lowest-priced: ………........  Highest-price:…………...… |  |  |
| Tortilla chips | ..................... |  |  |  |  |  |  |  | Lowest-priced: ………........  Highest-price:…………...… |  |  |
|  | ..................... |  |  |  |  |  |  |  | Lowest-priced: ………........  Highest-price:…………...… |  |  |
|  | ..................... |  |  |  |  |  |  |  | Lowest-priced: ………........  Highest-price:…………...… |  |  |
|  | ..................... |  |  |  |  |  |  |  | Lowest-priced: ………........  Highest-price:…………...… |  |  |

***** Product in this category with the greatest reduction in both fat and salt.

**Measure 11- Breakfast cereals**

Are **breakfast cereal** products available in this store? Yes□ No□

**Availability, prominence, and price**

| **Food item** | **Availability** | | **Shelf location** | **Shelf height (Cm)** | **Shelf length**  **(Cm)** | **Shelf depth (Cm)** | **Number of shelves** | **Price** | **weight** |  |
| --- | --- | --- | --- | --- | --- | --- | --- | --- | --- | --- |
|  | **Yes** | **No** |  |  |  |  |  |  |  |  |
| **Healthy options** | | | | | | | | | | |
| Breakfast cereals with reduced sugar or fat or plain breakfast cereals |  |  |  |  |  |  |  | Lowest-priced: …….....  Highest-price:……...… |  |  |
|  |  |  |  |  |  |  |  |  |  |  |
| **Unhealthy options** | | | | | | | | | | |
| Flavored breakfast cereal |  |  |  |  |  |  |  | Lowest-priced: …….....  Highest-price:……...… |  |  |
|  |  |  |  |  |  |  |  |  |  |  |

**Measure 12- Sweets**

Are **sweets** products available in this store? Yes□ No□

**Availability, prominence, and price**

| **Food item** | **Availability** | | **Shelf location** | **Shelf height (Cm)** | **Shelf length**  **(Cm)** | **Shelf depth (Cm)** | **Number of shelves** | **Price per Weight or Quantity** |
| --- | --- | --- | --- | --- | --- | --- | --- | --- |
|  | **Yes** | **No** |  |  |  |  |  |  |
| **Candy** |  |  |  |  |  |  |  | Lowest-priced: ………..............  Highest-price: ….….….........… |
| Cocoa and chocolate products |  |  |  |  |  |  |  | Lowest-priced: ………..............  Highest-price: ….….….........… |
| Jelly |  |  |  |  |  |  |  | Lowest-priced: ………..............  Highest-price: ….….….........… |
| Gum |  |  |  |  |  |  |  | Lowest-priced: ………..............  Highest-price: ….….….........… |
| Gummy candies |  |  |  |  |  |  |  | Lowest-priced: ………..............  Highest-price: ….….….........… |

**Measure 13- Sugar, sweeteners, and other types of simple sugars**

Are **Sugar, sweeteners, and other types of simple sugar** products available in this store? Yes□ No□

**Availability, prominence, and price**

| **Food item** | **Availability** | | **Shelf location** | **Shelf height (Cm)** | **Shelf length**  **(Cm)** | **Shelf depth (Cm)** | **Number of shelves** | **Price** | **Weight** |
| --- | --- | --- | --- | --- | --- | --- | --- | --- | --- |
|  | **Yes** | **No** |  |  |  |  |  |  |  |
| Sugar cubes |  |  |  |  |  |  |  | Lowest-priced: …............  Highest-price: …......… |  |
| Sugar |  |  |  |  |  |  |  | Lowest-priced: …............  Highest-price: …......… |  |
| Rock candy |  |  |  |  |  |  |  | Lowest-priced: …............  Highest-price: …......… |  |
| Honey |  |  |  |  |  |  |  | Lowest-priced: …............  Highest-price: …......… |  |
| Halva (tahini-based sweet) |  |  |  |  |  |  |  | Lowest-priced: …............  Highest-price: …......… |  |

**Measure 14- Rice, pasta, and legumes**

Are **Rice, pasta, and legume** products available in this store? Yes□ No□

**Availability, prominence, and price**

| **Food item** | | **Availability** | | **Shelf location** | **Shelf height (Cm)** | **Shelf length**  **(Cm)** | **Shelf depth (Cm)** | **Number of shelves** | **Price** | **weight** |
| --- | --- | --- | --- | --- | --- | --- | --- | --- | --- | --- |
|  |  | **Yes** | **No** |  |  |  |  |  |  |  |
| Rice | |  |  |  |  |  |  |  | Lowest-priced: ..........  Highest-price: ............ |  |
|  |  |  |  |  |  |  |  |  | Lowest-priced: ..........  Highest-price: ............ |  |
|  |  |  |  |  |  |  |  |  | Lowest-priced: ..........  Highest-price: ............ |  |
| Pasta | |  |  |  |  |  |  |  | Lowest-priced: ..........  Highest-price: ............ |  |
|  |  |  |  |  |  |  |  |  | Lowest-priced: ..........  Highest-price: ............ |  |
|  |  |  |  |  |  |  |  |  | Lowest-priced: ..........  Highest-price: ............ |  |
| Legumes | Chickpeas |  |  |  |  |  |  |  | Lowest-priced: ..........  Highest-price: ............ |  |
|  | Beans |  |  |  |  |  |  |  | Lowest-priced: ..........  Highest-price: ............ |  |
|  | Split peas |  |  |  |  |  |  |  | Lowest-priced: ..........  Highest-price: ............ |  |
|  | Mung beans |  |  |  |  |  |  |  | Lowest-priced: ..........  Highest-price: ............ |  |
|  | Lentils |  |  |  |  |  |  |  | Lowest-priced: ..........  Highest-price: ............ |  |
|  | Broad beans |  |  |  |  |  |  |  | Lowest-priced: ..........  Highest-price: ............ |  |
|  | Soybeans |  |  |  |  |  |  |  | Lowest-priced: ..........  Highest-price: ............ |  |
|  | Others: |  |  |  |  |  |  |  | Lowest-priced: ..........  Highest-price: ............ |  |

**Measure 15- Promotions**

1. **In-store advertising**

| Prize offer | Advertising character | Method of advertisement presentation | Size of the advertisement | Advertised food product - Food group | Row |
| --- | --- | --- | --- | --- | --- |
|  |  |  |  |  | 1 |
|  |  |  |  |  | 2 |
|  |  |  |  |  | 3 |
|  |  |  |  |  | 4 |
|  |  |  |  |  | 5 |
|  |  |  |  |  | 6 |
|  |  |  |  |  | 7 |
|  |  |  |  |  | 8 |
|  |  |  |  |  | 9 |
|  |  |  |  |  | 10 |

1. **Out-of-store advertising**

| Prize offer | Advertising character | Method of advertisement presentation | Size of the advertisement | Advertised food product - Food group | Row |
| --- | --- | --- | --- | --- | --- |
|  |  |  |  |  | 1 |
|  |  |  |  |  | 2 |
|  |  |  |  |  | 3 |
|  |  |  |  |  | 4 |
|  |  |  |  |  | 5 |
|  |  |  |  |  | 6 |
|  |  |  |  |  | 7 |
|  |  |  |  |  | 8 |
|  |  |  |  |  | 9 |
|  |  |  |  |  | 10 |

**Food Environment Assessment tool Protocol - Stores**

**Basic guidelines**

**This tool is designed for ranking the food environment of grocery stores, supermarkets, and similar retail outlets. There are other types of stores that may offer food products for sale, but they are not covered by this protocol and are considered as exclusion criteria (refer to the exclusion criteria section). These stores may be counted in the surveys but are not necessarily evaluated. It is important to note that depending on the objectives of your study in using this protocol, you may consider different exclusion criteria.**

**Exclusion criteria**

1. Stores that are not open to the public (such as stores related to specific institutions) or have an entry fee.
2. Stores that offer a limited variety of food items and do not fall into the category of grocery stores or supermarkets.
3. Vending machines are also excluded from this list.

**Aims**

**The main objective of this protocol is to collect information on comparable food items and products across different stores. Therefore, whenever possible, rank the items with the same brand or exactly as specified in this guide.**

**Obtaining permission and consent form**

Researchers must obtain approval from the supermarket or store manager before collecting data. Depending on the type of supermarket (independent or chain), approval from the headquarters may be required. It is recommended to first consult with the local store managers, and only if local managers do not have the authority to grant permission and approval, should you consult with the headquarters.

It is advisable to inform supermarket or store managers about the research via a letter (or in person). If present, an introduction letter, information sheet, and consent form (if applicable) should be provided to the store manager. It is recommended to minimize any potential disruption to the stores and supermarket's activities caused by the research. This may mean that data collection should be carried out during times of the day when there are fewer customers in the store.

**Time**

1. The evaluation of grocery stores should be conducted between 9 AM and 4 PM (during this time, the store's items for the day are stocked and not yet fully sold).
2. **The evaluation of stores and supermarkets should be conducted before 4:30 PM or after 6 PM (during these times, the stores are less crowded).**

**Assessment criteria in the protocol**

1. **Availability**

Shelf space is a highly sensitive criterion for assessing food availability in supermarkets. In this protocol, to evaluate the availability of specified food groups and products, shelf space (comprising the length and depth of shelves) and the number of shelves are used. Measurements should be recorded in a table, and then the number of shelves allocated to the specified products should be measured for each recorded distance. If a food item is displayed or offered in more than one location within the store, each location is measured separately and then summed together after all measurements are completed. Note that for assessing availability, analyzing it, and comparing between different groups and healthy and unhealthy foods, the total floor space of the supermarket/store is also needed.

1. **Prominence**

To assess the visibility of food products in supermarkets, the placement of products and the weight of these locations at different points in the store are examined. The visibility of products is classified based on customer exposure to various areas as high, medium, or low. For example, a product located near the checkout counter is more likely to attract attention than a product at the far end of the store. Additionally, the height of shelves from the ground is evaluated to assess the visibility of food items for specific age groups, considering their height and interaction with the food products. It should be noted that When measuring the height of the shelf from the ground, if multiple shelves with the desired food product are present, use the lowest shelf in terms of height as the reference and record its height from the ground.

1. **Price**

In this protocol, the price of food items is measured based on their weight or volume. The advantage of this method is that it allows for comparison of the prices of all food items in terms of grams or milliliters of that food product with other items in later stages. This method can also be easily used to compare the average prices of healthy and unhealthy groups and various food items. Price comparison between healthy and unhealthy groups is done by calculating the average lowest price of all items in each group and then comparing these averages.

Note that for all food items, you should record the consumer price and the weight or volume from the product packaging or label. If the price is not available, inquire with a cashier or customer service. Do not use discounted (sale) prices unless it is the only price available, and be sure to note in the table that the stated price is a “discounted price.

1. **Promotion**

**In two phases, the evaluation of in-store and out-of-store advertisements is carried out, and all food advertisements inside and outside the store are assessed. The evaluation of advertisements is performed using a table consisting of items such as advertisement size, advertising character, prize offer, and so on, in the final stage.**

**Data collection**

At the top of the first page, include the following information:

1. **Name of the evaluator**
2. **Store name**
3. **Type of store**
   1. **Kiosk:** Very small retail units, typically open-fronted or with minimal enclosed space, primarily offering a limited range of convenience items. These outlets had no aisles or shelving for food or non-food products.
   2. **Small supermarket:** Small enclosed shops without formal aisles but larger and more structured than kiosks. These stores displayed a broader range of convenience products and were distinctly different from kiosks in terms of layout and size.
   3. **Medium/Large supermarket:** Stores featuring more than 2 aisles, offering a wider selection of food and non-food items.
   4. **Other:** Includes specialty stores for a specific product, such as butcher shops, greengrocers, health food stores, etc.
4. **Date of visit to the store**
5. **Start time (when you enter the store)**
6. **End time (when the evaluation is completed)**
7. **Number of cash registers in the store (including any register where transactions are processed, even if no cashier is present at the time of visit)**
8. **Number of aisles:** Refers to the number of aisles between the shelves in the store.
9. **Store floor area**
10. **Neighborhood name:** The name of the neighborhood where the store is located.
11. **Obtained consent form from the store owner/manager**

**Measure 1- Milk and dairy**

1. Find the dairy section in the store. If dairy products (milk, yogurt, buttermilk, or cheese) are not sold in the store, select "No" for this section and proceed to the next food group. If dairy products are available, select "Yes" and continue.

**Availability and prominence**

1. For each dairy item listed (milk, yogurt, buttermilk, and cheese), record the available sizes in the store in the first column (e.g., 1-liter milk, 250-milliliter milk, or 500-gram cheese).
2. Specify the location of these groups within the store according to the table at the end of the protocol (refer to Appendix 1).
3. In the following columns, record the length, height, depth, and number of shelves related to each of these sizes. Then, in the next four columns, indicate the percentage of these shelves dedicated to each of the specified categories (e.g., low-fat or low-sodium, full-fat, flavored, and plant-based).

**Price**

1. In the next table, for each of the specified dairy items (milk, yogurt, buttermilk, and cheese), the healthy and unhealthy options are listed. In the volume column, select only one uniform size (preferably 1 liter) for all food items. In the price column, record the highest and lowest prices of that product available in the store (e.g., note the highest and lowest prices for 1-liter low-fat or semi-skimmed milk in the relevant row).

**Measure 2 - Fruits**

**Healthy Options (Fresh fruits & Frozen fruits)**

1. Find the fresh or frozen fruits section in the store. If none of these items are available in the store, mark "No" for the first question and proceed to the next section. If available, mark "Yes" and continue.

**Availability and prominence**

1. In the second column, note the type of fruit available in the store. For example, various types of apples (yellow, green, red, etc.) or oranges (common, Thomson, etc.). Specify and record the type of fruit. If the fruit type is not specified or cannot be determined, record it as "common".
2. If the specified fruit is available, mark "Yes". If it is not available, mark "No".
3. Measure and record the location of each type of fruit's shelf in the store, along with the length, height, depth, and number of shelves.
4. Determine and mark the quality of the fruits available in the store based on your assessment (high or low quality):
   - High Quality: Best condition, excellent quality, good color, fresh, firm, and clean.
   - Low Quality: Bruised, old appearance, stained, dry, overripe, dented, scratched, or broken surfaces, wrinkling, moldy, or overly soft.

Quality assessment should be based on the majority of available fruits. If it's difficult to determine the quality, mark "UA" and explain in the notes. If mixed fruits are available, note in the description which fruits are included in the mixed basket or package.

**Price**

1. Record the price of the fruit based on the price tag. Record the price per kilogram. If prices are listed by the number of fruits, record both the quantity and price. If fruits are packaged in a container, note the weight of the container and its price (be sure to specify whether the price is for the entire container or per kilogram).

**Unhealthy Options (Jam, Fruit Leather, Dried Fruits, and Fruit compote)**

1. Find the relevant section in the store. If none of these items are available in the store, mark "No" for the first question and proceed to the next section. If available, mark "Yes" and continue.

**Availability and prominence**

1. Record the different weights available for each specified item (jam, fruit leather, etc.) in the store.
2. If the item is available, mark "Yes". If it is not available, mark "No".
3. Measure and record the location of each item's shelf in the store, along with the length, height, depth, and number of shelves.

**Price**

1. Note the lowest and highest prices for the specified product by weight (e.g., the price of the cheapest and most expensive 300-gram jam).
2. Specify the type of product (e.g., if the cheapest 300-gram jam available is carrot jam, record "carrot jam" in this column).

**Measure 3- Vegetables**

**Healthy Options (Fresh vegetables and Frozen vegetables)**

1. Find the fresh and frozen vegetables section in the store. If fresh vegetables are not available, mark "No" for the first question and proceed to the next food group. If any are available, mark "Yes" and continue.

**Availability and prominence**

1. If any of the listed vegetables are available, mark "Yes". If not, mark "No".
2. If specific types of each vegetable are available in the store, note them in the next column. Then, measure and record the location of each item’s shelf in the store, along with the length, height, depth, and number of shelves.
3. Determine and mark the quality of the vegetables offered in the store based on your assessment (high or low quality):
   - High Quality: Best condition, excellent quality, good color, fresh, firm, and clean.
   - Low Quality: Bruised, old appearance, stained, dry, overripe, dented, scratched, or broken surfaces, wrinkling, moldy, or overly soft.

Quality assessment should be based on the majority of available vegetables. If it is difficult to determine the quality, mark "UA" and explain in the notes.

**Price**

1. Record the lowest and highest price for each available vegetable based on the price tag. Record the price per kilogram. If the vegetables are packaged in a container, note the weight of the container and its price (be sure to specify whether the price is for the entire container or per kilogram).

**Unhealthy Options (Canned vegetables and fried vegetables)**

1. Find the relevant section in the store. If the listed vegetables are not available, mark "No" for the first question and proceed to the next food group. If any are available, mark "Yes" and continue.

**Availability and prominence**

1. Record the different weights available for each vegetable item. Mark the availability of each item. If not available, mark "No".
2. Measure and record the location of each item’s shelf in the store, along with the length, height, depth, and number of shelves.
3. Determine and mark the quality of the product offered in the store based on your assessment (high or low quality):
   - High Quality: Best condition, excellent quality, good color, fresh, firm, and clean.
   - Low Quality: Bruised, old appearance, stained, dry, overripe, dented, scratched, or broken surfaces, wrinkling, moldy, or overly soft.
   - Quality assessment should be based on the majority of available processed vegetables. If it is difficult to determine the quality, mark "UA" and explain in the notes.

**Price**

1. Record the lowest and highest price for each processed vegetable item based on the price tag. Record the price per kilogram. If the vegetables are packaged in a container, note the weight of the container and its price (be sure to specify whether the price is for the entire container or per kilogram).

**Measure 4- Meats**

**Availability and prominence**

1. Find the section for meats or eggs in the store. If the store does not sell packaged meat, go to the store's butcher section and examine the meat there. If meat or eggs are not available in the store, mark "No" for the first question and proceed to the next item. If available, mark "Yes" and continue.
2. For healthy options, find chicken, turkey or other poultry, fish, shrimp, or eggs. If any of these are available, mark "Yes".
3. For each available option, note the location of the shelf, the length, height, depth, and number of shelves.
4. For regular options, find regular beef, regular lamb, ground beef, or mixed ground meat and repeat the previous steps if available.

**Price**

1. For each item that was available and recorded in the store, note the lowest and highest price per kilogram. (Ensure that prices are per kilogram. If prices are given per package weight, convert them to per kilogram prices.)

**Measure 5- Hot dog, sausage, and deli meat**

**Availability and prominence**

1. Find the section for hot dogs, sausages, and deli meats in the store. If none of these items are available, mark "No" for the first question and proceed to the next section. If any of these items are available, mark "Yes" and continue.
2. For healthy options, look for healthy plant-based products with reduces levels of salt or fat. If available, mark "Yes" and record the length, height, depth, and number of shelves for each item.
3. For unhealthy options, look for unhealthy plant-based (with high level of fat or salt) and regular options. If not available, mark "No"; if available, mark "Yes" and record the length, height, depth, and number of shelves for these items.

**Note:** Although vegan sausages are plant-based and often considered more sustainable, their healthiness can vary considerably depending on their nutrient composition—particularly in terms of salt and fat content. Given this variability, the checklist allows for case-by-case classification of vegan sausages based on the product’s traffic light nutrition label. Evaluators are instructed to examine the label and determine whether the item meets the criteria to be considered healthy or unhealthy. This approach ensures greater flexibility and accuracy in classification.

**Price**

1. For each item that was available and recorded, note the lowest and highest price per kilogram. (Ensure that prices are per kilogram. If prices are given per package weight, convert them to price per kilogram.)

**Measure 6- Ready-to-eat food**

1. Locate the section for ready-to-eat foods in the store, which includes items like ready-made stews, canned fish, various canned foods, sandwiches, burgers, falafel, nuggets, lasagna, pizza, fried fillets, kebabs, etc. If prepared foods are not available, mark "No" for the first question and proceed to the next section. If available, mark "Yes" and continue.

**Availability and prominence**

1. In the first column, identify and record the types of ready-to-eat foods available in the store (e.g., stew, canned fish, canned meals, etc.). For healthy options, look for ready-to-eat foods with a fat content of 10 grams or less. If any of these items are available, mark “Yes” and proceed.
2. In the following columns, record the location, length, height, depth, and number of shelves for each weight of prepared foods.
3. For unhealthy options, look for ready-to-eat foods with fat content greater than 10 grams. If not available, mark "No"; if available, mark "Yes" and record the length, height, depth, and number of shelves for these items.

**Price**

1. For each type of ready-to-eat food, identify the most expensive and the least expensive options available, and record them along with the weight of the product.

**Measure 7- Cake, cookie, and biscuit**

**Availability and prominence**

1. Locate the section for cakes, cookies, and biscuits in the store. If these items are not available, mark "No" for the first question and proceed to the next section. If available, mark "Yes" and continue.
2. For healthy options, look for whole-grain cakes, cookies, rice cakes, or their alternatives (diet biscuits (sugar-free or reduced sugar), diet cakes (sugar-free or reduced sugar), and diet cookies (sugar-free or reduced sugar)). If any of these are available, mark "Yes" for the access section and record the location, length, height, depth, and number of shelves for each item.
3. For unhealthy options, look for plain cakes, cookies, or flavored biscuits (without whole grains). If these items are not available, mark "No"; if available, mark "Yes" and record the length, height, depth, and number of shelves for each item.

**Price**

1. For both healthy and unhealthy options, identify and record the most expensive and least expensive types of each product available in the store, including their weight. For example, if whole-grain cakes are available, note the cheapest and most expensive type, along with its weight.

**Measure 8- Beverages**

**Availability and prominence, and price-section 1**

1. Locate the beverages section in the store. If none of the specified beverages are available, mark "No" for the first question and proceed to the next section. If any are available, mark "Yes" and continue.

**Healthy Options**

1. Look for water in the store. Record the different volumes of water available in the first column. For each, mark "Yes" if available, and note the location, length, height, depth, and number of shelves. Record the lowest and highest price for each volume available in the store (e.g., the price of the cheapest and most expensive 1-liter bottle of water).

**Unhealthy Options**

1. Look for listed items. If these items are not available, mark "No"; if available, mark "Yes" and record the location, length, height, depth, and number of shelves for each item. Record the lowest and highest price for each volume available in the store, similar to the healthy options.

**Availability and prominence, and price – Section 2**

1. For healthy options, look for natural fruit juice with more than 25% fruit content in the store. Record the different volumes of these beverages available in the first column. For each, mark "Yes" if available, and note the location, length, height, depth, and number of shelves. Record the lowest and highest price for each volume available in the store (e.g., the price of the cheapest and most expensive 100% natural 1-liter fruit juice).
2. For unhealthy options, look for beverages containing fruit juice with 25% or less fruit content. If these items are not available, mark "No"; if available, mark "Yes" and record the location, length, height, depth, and number of shelves for each item. Record the lowest and highest price for each volume available in the store, similar to the healthy options.

**Measure 9- Bread and bakery products**

**Bread Definitions and Evaluation**

**A. Healthy Options:** All kinds of bread made from whole-grain or multi-grain flour, or breads without added sugar and with reduced salt

**B. Unhealthy Options:** All kinds of bread made from refined flour, with added sugar and/or high salt content

**Availability and prominence, and price**

1. **Locate the bread section in the store. If bread is not available, mark "No" for the first question and proceed to the next section. If bread is available, mark "Yes" and continue.**
2. **For healthy options, identify bakery products and bread made** from whole-grain or multi-grain flour, or breads without added sugar and with reduced salt**. Record the type of bread available in the first column, and note the location, length, height, depth, and number of shelves for each.**
3. **For unhealthy options, identify bakery products and bread** made from refined flour, with added sugar and/or high salt content**. Record the type of bread available in the first column, and note the location, length, height, depth, and number of shelves for each.**
4. **For each product listed, record the package weight and its price.**

**Measure 10- Chips and cheese puffs**

**Availability and prominence, and price**

1. **Locate the section for chips and puffed snacks in the store. If these items are not available, mark "No" for the first question and proceed to the next section. If these items are available, mark "Yes" and continue.**
2. **Based on the context and conditions of the research, for healthy options, look for the products in this category which have the greatest reduction in both fat and salt. Record the different weights available for this product, note the location, length, height, depth, and number of shelves related to this product.**
3. **For unhealthy options, look for salted potato chips, puffed corn snacks (**cheese puffs**), popcorn, and tortilla chips. Record the different weights available for these products, and note the location, length, height, depth, and number of shelves related to each product.**
4. **Record the lowest and highest prices associated with each weight among the available products in the store.**
5. **In the last column, specify the type of product that has the highest and lowest prices.**

**Measure 11- Breakfast cereals**

**Availability and prominence, and price**

1. **Go to the breakfast cereal section in the store. If breakfast cereals are not available, mark "No" for the first question and move on to the next section. If breakfast cereals are available, mark "Yes" and continue.**
2. **For the healthy option, look for breakfast cereals with reduced sugar or fat or plain breakfast cereals. You may also choose cereals labeled as low-calorie or low-sugar. If these options are available, mark "Yes" and record the location, length, height, depth, and number of shelves for these products.**
3. **For the unhealthy option, look for flavored breakfast cereal. If these options are available, mark "Yes" and record the location, length, height, depth, and number of shelves for these products.**
4. **Record the highest and lowest prices among the healthy and unhealthy products, and note the packaging weight in the corresponding column.**

**Measure 12- Sweets**

**Availability and prominence, and price**

1. **Go to the sweets section in the store. If sweets are not available, mark "No" for the first question and move on to the next section. If sweets are available, mark "Yes" and continue.**
2. **Evaluate the availability of the following items: candies, cocoa and chocolate bars, jelly, gum, and gummy candies. If any of these items are available, mark "Yes" and record the location, length, height, depth, and number of shelves for each product.**
3. **Record the highest and lowest prices for each available item in the section and note the weight in the corresponding column.**

**Measure 13- Sugar, sweeteners, and other types of simple sugars**

**Availability and prominence, and Price**

1. **Locate the section for sugar, simple sugars, and similar products in the store. If none of these products are available, mark "No" for the first question and move on to the next section. If any of these products are available, mark "Yes" and continue.**
2. **For each available product, mark its availability and record the location, length, height, depth, and number of shelves dedicated to that product in the subsequent columns.**
3. **Record the highest and lowest prices available for each product, along with the weight of the product, in the corresponding columns.**

**Measure 14- Rice, pasta, and legumes**

**Availability and prominence, and Price**

1. **Go to the section for rice, pasta, and legumes in the store. If any of these products are not available, mark "No" and move on to the next section. If they are available, mark "Yes" and continue.**
2. **Record the location, length, height, depth, and number of shelves dedicated to each of these products (rice, pasta, and legumes) in the corresponding columns.**
3. **Note the highest and lowest prices available for each product, along with the weight of the product, in the subsequent columns.**

**Measure 15- Promotions**

The collection and extraction of information related to advertisements are conducted in two phases: evaluation of in-store advertisements and evaluation of out-store advertisements:

1. **In-Store Advertisements:** Includes all advertisements that are installed or placed inside the store's space.
2. **Out-Store Advertisements:** Includes all advertisements that are installed or placed behind the glass or in the vicinity of the store.

Evaluate and review each advertisement, analyze the information based on the available advertisement details, and record it in the relevant tables. Enter the information into the tables according to the specified codes in the tables below.

| Record the advertised food product alongside its corresponding group (based on the groups specified in the protocol). For example: "Soda - Beverage Group" or "Chickpeas - Rice, Pasta, and Legumes Group | **Advertised Food Product - Food Group** |
| --- | --- |
| **Code 1** → Small (smaller than 1.9 × 1.3 meters)  **Code 2** → Medium (between 1.9 × 1.3 meters and 2.5 × 0.2 meters)  **Code 3** → Large (larger than 2.5 × 0.2 meters) | **Ad Size** |
| **Code 1** → Billboard  **Code 2** → Poster or Banner  **Code 3** → Stand  **Code 4** → Painted  **Code 5** → LED Advertising Display  **Code 6** → Store Fixtures (e.g., on food shelves outside the store or store awnings)  **Code 7** → Other (please specify) | **Ad Presentation Method** |
| **Code 0** → No Advertiser Character  **Code 1** → Brand or Product Mascot (e.g., cartoon characters like the monkey on Cheetos products or the Daily Cow character)  **Code 2** → Cartoon Character  **Code 3** → Amateur Athlete (an ordinary person engaged in sports)  **Code 4** → Famous or Well-Known Athlete/Sports Team  **Code 5** → Non-Sport/Event Related (historical events, festivals, or religious holidays like Nowruz or Yalda Night)  **Code 6** → Child-Oriented Product (e.g., featuring images of children)  **Code 7** → Awards Received (e.g., Best Food Award of 2020 as voted by consumers)  **Code 8** → Reference to a Sports Event in the Advertisement | **Advertiser Character** |
| **Code 0** → No Prize Offer  **Code 1** → Downloadable Games or Apps  **Code 2** → Contests  **Code 3** → Buy 2 Get 3 or Similar Offers  **Code 4** → Limited Quantity Product  **Code 5** → Charity Donation  **Code 6** → Gift  **Code 7** → Price Discount  **Code 8** → Exclusive for Loyal Customers  **Code 9** → Entry into a Raffle | **Prize Offer** |

**Data Reliability Evaluation**

1. **Training**
   All data collectors must receive training before data collection begins. Their initial data collection should be supervised, and measurements should be reviewed. In cases of discrepancies, additional training and explanations must be provided.
2. **Reliability Check**
   **Access:** For approximately 10% of supermarkets, the data must be re-evaluated during a secondary assessment by another researcher. This check should be conducted as soon as possible, preferably simultaneously with the initial data collection and after the primary data collection.

One aspect of this involves calculating the percentage agreement in shelf space evaluation (length × depth) between two evaluators, which is computed as follows:

$\frac{Number of Shelf Spaces with Agreement}{Number of Shelf Spaces with Disagreement+Number of Shelf Spaces with Agreement}\times100$

In this section, other evaluations include:

1. **Number of Supermarkets Included in the Study**
2. **Percentage Agreement Between Evaluators for the Number of Shelves**
3. **Percentage Agreement Between Evaluators for Shelf Placement**
4. **Percentage Agreement Between Evaluators for the Number of Advertisements Found in the Store**
5. **Percentage Agreement Between Evaluators for the Score Achieved by Each Store**

**Data Analysis**

**Approach One:** Based on scoring stores, each store or supermarket is assigned a specific score according to the data collected. Higher scores are associated with healthier stores, while lower scores are associated with less healthy stores. Comparing stores can be done by comparing these scores to determine their healthiness. Additionally, these scores can be computed and compared based on availability, price, or quality scores.

**Approach Two:** Each evaluation criterion in the guidelines (availability, price, etc.) is analyzed independently:

1. **Availability and prominence:**
   - For each type of food item, determine the shelf space (length × depth) (or square centimeters) for each product type in different placement locations. Then, categorize the locations as low, medium, or high prominence based on the attached table.
   - Sum the shelf space (length × number of shelves) (or square centimeters) in each category (low, medium, high visibility) to get the total shelf space for each category. Metrics to calculate include:
     - Shelf space for each type of healthy product
     - Shelf space for each type of healthy product by placement location (low, medium, high visibility)
     - Shelf space for each type of unhealthy product
     - Shelf space for each type of unhealthy product by placement location (low, medium, high visibility)
     - Total shelf space for all healthy products
     - Total shelf space for all healthy products by placement location (low, medium, high visibility)
     - Total shelf space for all unhealthy products
     - Total shelf space for all unhealthy products by placement location (low, medium, high visibility)
     - Ratio of shelf space for healthy products to unhealthy products
     - Ratio of shelf space for healthy products to unhealthy products by placement location (low, medium, high visibility)
     - Ratio of shelf space for each type of healthy product to store area
     - Ratio of total shelf space for healthy food items to store area
     - Ratio of shelf space for each type of unhealthy product to store area
     - Ratio of total shelf space for unhealthy food items to store area
2. **Advertisements:**
   - Metrics to calculate include:
     - Ratio of the number of healthy to unhealthy advertisements that offer discounts or prizes.
     - Ratio of the number of healthy to unhealthy advertisements featuring specific characters or spokespersons.
     - Ratio of food advertisements categorized by main food categories.
3. **Price:**
   - Initially, calculate the price of all food items per 100 grams or 100 milliliters. Metrics to calculate include:
     - Average price of healthy food items in each group
     - Average price of unhealthy food items in each group
     - Difference between the highest and lowest price for each food item
     - Difference between the average of the highest and lowest prices for healthy and unhealthy food groups

**Appendix 1 - Shelf Placement Locations Inside the Store:**

| **definition** | **Prominence** | **Shelf placement** | **Code** |
| --- | --- | --- | --- |
| Area within 3 meters in front of the store entrance | **Medium** | **Entrance** | 1 |
| When shelves are extended along the front (where the checkouts are) and the back of the store, the section of the shelves facing towards the front of the store is referred to as Shelf Front 1. If the store has shelves extending along the left and right sides, Shelf Front 1 includes the shelves that are oriented towards the center of the store. | High | **Endcap 1** | 2 |
| When shelves are extended along the front (where the checkouts are) and the back of the store, the section of the shelves facing towards the back of the store is referred to as Shelf Rear 2. If the store has shelves extending along the left and right sides, Shelf Rear 2 includes the shelves that are oriented towards the outer sides of the store | **Medium** | **Endcap 2** | 3 |
| **Includes all shelves located in the store's aisles. This also includes permanent circular or round shelves.** | **Medium** | **Aisle** | 4 |
| Includes all the edges around the store that are attached to the walls. | Low | Edge | 5 |
| **Includes non-permanent and movable shelving. This category encompasses tables, temporary racks, and bins (if these items are permanent, they are classified under aisle shelves).** | **Medium** | Island | 6 |
| **The area directly beside and in front of the checkout counters.** | High | Checkout Side | 7 |
| **Includes the area and shelves near the checkout where customers encounter them before reaching the checkout counter.** | High | Checkout End | 8 |

**Comparison and Scoring**

1. **Availability, price and quality**

| **Food group** | **Availability** | **Price** | **Quality** | **Max. score** |
| --- | --- | --- | --- | --- |
| **Milk and dairy** | **2 points** → Healthier options are available for all items in the dairy group.  **1 point** → Healthier options are available for at least one item in the dairy group.  **0 points**→ Healthier options are not available for any item in the dairy group. | **2 points** → The average price of the cheapest healthier options per milliliter for all items in the dairy group is less than that of the unhealthy options.  **1 point** → The average price of the cheapest healthier options per milliliter for all items in the dairy group is equal to the average price of the unhealthy options.  **0 points** → The average price of the cheapest healthier per milliliter for all items in the dairy group is more than that of the unhealthy options. |  | **5 points** |
|  | **1 point** → The percentage of availability of healthier for all items averages more than 50% of the total shelf space dedicated to dairy products.  **0 points** → The percentage of availability of healthier options for all items averages 50% or less of the total shelf space dedicated to dairy products. |  |  |  |
| **Fruits** | **2 points** → If healthier options occupy a greater proportion of shelf space compared to unhealthy options.  **1 point** → If healthier options and unhealthy options occupy an equal proportion of shelf space.  **0 points** → If unhealthy options occupy a greater proportion of shelf space compared to healthier options. | **2 points** → The average price of the cheapest healthy options per gram is less than that of unhealthy options.  **1 point** → The average price of the cheapest healthy options per gram is equal to that of unhealthy options.  **0 points** → The average price of the cheapest healthy options per gram is more than that of unhealthy options. | **3 points** → More than 75% of the available fruits are of high quality.  **2 points** → 74-50% of the available fruits are of high quality.  **1 point** → 49-25% of the available fruits are of high quality. | **7 points** |
| **Vegetables** | **2 points** → If healthier options occupy a greater proportion of shelf space compared to unhealthy options.  **1 point** → If healthier options and unhealthy options occupy an equal proportion of shelf space.  **0 points** → If unhealthy options occupy a greater proportion of shelf space compared to healthier options. | **2 points** → The average price of the cheapest healthy options per gram is less than that of unhealthy options.  **1 point** → The average price of the cheapest healthy options per gram is equal to that of unhealthy options.  **0 points** → The average price of the cheapest healthy options per gram is more than that of unhealthy options. | **3 points** → More than 75% of the available fruits are of high quality.  **2 points** → 74-50% of the available fruits are of high quality.  **1 point** → 49-25% of the available fruits are of high quality. | **7 points** |
| **Meats** | **2 points** → If healthier options occupy a greater proportion of shelf space compared to unhealthy options.  **1 point** → If healthier options and unhealthy options occupy an equal proportion of shelf space.  **0 points** → If unhealthy options occupy a greater proportion of shelf space compared to healthier options. | **2 points** → The average price of the cheapest healthy options per gram is less than that of unhealthy options.  **1 point** → The average price of the cheapest healthy options per gram is equal to that of unhealthy options.  **0 points** → The average price of the cheapest healthy options per gram is more than that of unhealthy options. |  | **4 points** |
| **Hot dog, sausage, and deli meat** | **2 points** → If healthier options occupy a higher percentage of the shelves compared to unhealthy options or their alternatives.  **1 point** → If healthier options occupy an equal percentage of the shelves compared to unhealthy options or their alternatives.  **0 points** → If unhealthy options occupy a higher percentage of the shelves compared to healthier options. | **2 points** → If the average price of healthier options per gram is less than that of unhealthy options.  **1 point** → If the average price of healthier options per gram is equal to that of unhealthy options.  **0 points** → If the average price of healthier option per gram is higher than that of unhealthy options. |  | **4 points** |
| **Ready-to-eat food** | **2 points** → If healthier options occupy a greater proportion of shelf space compared to unhealthy options.  **1 point** → If healthier options and unhealthy options occupy an equal proportion of shelf space.  **0 points** → If unhealthy options occupy a greater proportion of shelf space compared to healthier options. | **2 points** → The average price of the cheapest healthy options per gram is less than that of unhealthy options.  **1 point** → The average price of the cheapest healthy options per gram is equal to that of unhealthy options.  **0 points** → The average price of the cheapest healthy options per gram is more than that of unhealthy options. |  | **4 points** |
| **Cake, cookie, and biscuit** | **2 points** → If healthier options occupy a greater proportion of shelf space compared to unhealthy options.  **1 point** → If healthier options and unhealthy options occupy an equal proportion of shelf space.  **0 points** → If unhealthy options occupy a greater proportion of shelf space compared to healthier options. | **2 points** → The average price of the cheapest healthy options per gram is less than that of unhealthy options.  **1 point** → The average price of the cheapest healthy options per gram is equal to that of unhealthy options.  **0 points** → The average price of the cheapest healthy options per gram is more than that of unhealthy options. |  | **4 points** |
| **Beverages** | **2 points** → If the total shelf space occupied by healthier options in both sections is greater than that occupied by unhealthy options in both sections.  **1 point** → If the total shelf space occupied by healthier options in both sections is equal to that occupied by unhealthy options in both sections.  **0 points** → If the total shelf space occupied by healthier options in both sections is less than that occupied by unhealthy options in both sections. | **2 points** → If the average price of the cheapest healthier options in both sections per milliliter is less than the average price of the cheapest unhealthy options in both sections.  **1 point** → If the average price of the cheapest healthier options in both sections per milliliter is equal to the average price of the cheapest unhealthy options in both sections.  **0 points** → If the average price of the cheapest healthier options in both sections per milliliter is higher than the average price of the cheapest unhealthy options in both sections. |  | **4 points** |
| **Bread and bakery products** | **2 points** → If healthier options occupy a greater proportion of the shelves compared to unhealthy options.  **1 point** → If healthier options occupy an equal proportion of the shelves compared to unhealthy options.  **0 points** → If healthier options occupy a lesser proportion of the shelves compared to unhealthy options. | **2 points** → If the average price of the cheapest healthy options per gram is less than the average price of the cheapest unhealthy options.  **1 point** → If the average price of the cheapest healthy options per gram is equal to the average price of the cheapest unhealthy options.  **0 points** → If the average price of the cheapest healthy options per gram is greater than the average price of the cheapest unhealthy options. |  | **4 points** |
| **Chips and cheese puffs** | **2 points** → If healthier options occupy a greater proportion of the shelves compared to unhealthy options.  **1 point** → If healthier options occupy an equal proportion of the shelves compared to unhealthy options.  **0 points** → If healthier options occupy a lesser proportion of the shelves compared to unhealthy options. | **2 points** → If the average price of the cheapest healthy options per gram is less than the average price of the cheapest unhealthy options.  **1 point** → If the average price of the cheapest healthy options per gram is equal to the average price of the cheapest unhealthy options.  **0 points** → If the average price of the cheapest healthy options per gram is greater than the average price of the cheapest unhealthy options. |  | **4 points** |
| **Breakfast cereals** | **2 points** → If healthier options occupy a greater proportion of shelf space compared to unhealthy options.  **1 point** → If healthier options and unhealthy options occupy an equal proportion of shelf space.  **0 points** → If unhealthy options occupy a greater proportion of shelf space compared to healthier options. | **2 points** → The average price of the cheapest healthy options per gram is less than that of unhealthy options.  **1 point** → The average price of the cheapest healthy options per gram is equal to that of unhealthy options.  **0 points** → The average price of the cheapest healthy options per gram is more than that of unhealthy options. |  | **4 points** |
| **sweets** | **2 points** → If none of the items are available in the store.  **1 point** → If 3 or fewer items are available in the store.  **0 points** → If more than 4 items are available in the store. |  |  | **2 points** |
| **Rice, pasta, and legumes** | **2 points** → If more than 7 items are available.  **1 point** → If 1 to 6 items are available.  **0 points** → If none of the items are available. |  |  | **2 points** |
| **Sugar, sweeteners, and other types of simple sugars** | **2 points** → If none of the items are available in the store.  **1 point** → If 3 or fewer items are available in the store.  **0 points** → If more than 4 items are available in the store. |  |  | **2 points** |

1. **Advertisements**
   The comparison of the number of healthy and unhealthy advertisements inside and outside the store is done as follows:
   1. **Inside the Store**:
      The criteria for classifying the advertised products as healthy or unhealthy are based on the list of harmful products and the nutrient profile model for marketing of food and non-alcoholic beverages to children in the Eastern Mediterranean region by the World Health Organization. Based on these criteria, classify the advertisements into healthy and unhealthy categories.
   2. **Outside the Store**:
      The criteria for classifying the advertised products as healthy or unhealthy are based on the list of healthy and unhealthy products as defined in the INFORMAS advertising protocol. Based on these criteria, classify the advertisements into healthy and unhealthy categories.

Report the number of healthy and unhealthy advertisements separately for inside and outside the store.

**Comparing Stores by Scores**
Stores can be compared based on each dimension evaluated in the protocol (availability, price, and quality). For an overall comparison, sum the scores for each dimension for each store and compare the total scores of the stores. A score closer to 57 indicates better healthiness of the store, while a score closer to 0 indicates poorer healthiness.

- **Availability Score**: From 0 to 29 points
- **Price Score**: From 0 to 22 points
- **Quality Score**: From 0 to 6 points
- **Total Score**: From 0 to 57 points

**Advertisements**: Scores are not assigned. Reports are based solely on the number of healthy and unhealthy advertisements for each store.
